# A HILIC‐IM‐MS‐Based Pharmacometabodynamic Study of the Effects of Orally Administered Gefitinib on the Polar Urinary Metabolic Phenotypes of C57Bl6 Mice

**DOI:** 10.1002/jssc.70163

**Published:** 2025-05-06

**Authors:** Adam King, Lee A. Gethings, Robert S. Plumb, Ian D. Wilson

**Affiliations:** ^1^ Waters Corporation Wilmslow UK; ^2^ Waters Corporation Milford Massachusetts USA; ^3^ Division of Systems Medicine Department of Metabolism Digestion and Reproduction Imperial College, Burlington Danes Building London UK

**Keywords:** metabolic pathways, metabolite identification, polar metabolome, urinary metabotypes

## Abstract

The effects of the anilinoquinazoline tyrosine kinase inhibitor (TKI) gefitinib on the polar urinary metabolome of mice following oral administration (50 mg/kg) were studied over the 24 h period post dose. Analysis was performed using a hydrophilic interaction liquid chromatographic separation (HILIC) hyphenated to cyclic ion mobility (cIM) and mass spectrometry (MS). This investigation revealed numerous time‐dependent changes in the polar urinary metabolic phenotype of gefitinib‐dosed mice that mirrored the plasma concentrations and urinary excretion of the drug and its metabolites. These changes showed both relative increases and decreases in the amounts of various endogenous metabolites, including 8‐hydroxydeoxyguanosine, thymidine, acetylcarnitine, isobutyrylcarnitine, myristoylglycine, 3‐phenylpropionylglycine, cyclic AMP, 19‐oxotestosterone, and 3′‐asparagine‐AMP. These changes were generally seen to be greatest at the time of the highest plasma and urinary concentrations of gefitinib. The concordance of these effects on the urinary metabolome with plasma/urine drug concentrations strongly implies a range of pharmacometabodynamic effects pointing to mechanism‐based regulation of a number of endogenous metabolic pathways by gefitinib.

## 1 Introduction

The use of untargeted metabolic profiling, or metabolic phenotyping (“metabotyping”), as employed in metabonomics/metabolomics, offers the opportunity to better understand the biochemical processes underlying physiological change, genetic modification, disease, toxicity, and response to treatment, etc. [1, 2, 3]. Previous studies have shown how the resulting metabotypes can be used to predict the therapeutic outcome of drug administration, or the likelihood of a toxic response to drug treatment based on pre‐dose metabolite profiles. This pharmacometabonomic approach [4, 5], also termed pharmacometabolomics [6], has been an area of much interest as an enabler of personalized medicine for those advocating such approaches, (see e.g., [7] for a recent review of the topic). In research on drugs or toxins, there is clearly considerable potential to use this type of metabolic profiling to validate the metabolic pathways expected to be affected by bioactive compounds. In addition, the use of such untargeted metabolite profiling may also be used to explore the unintended/unexpected consequences of drug administration on the metabolome via the detection of changes in the metabolic phenotypes. A knowledge of the pathways affected by drug treatment may then provide insights into the mechanism(s) of action (MOA) resulting in pharmacological/toxicological effects. By highlighting these changes, such analysis has the potential to afford mechanistic insights into the system‐wide effects of drug administration. The detection of any unexpected “off‐target” effects in this way may help to explain toxicity and even suggest alternative pharmacological targets, enabling the “repurposing” of compounds for completely different therapeutic conditions [8]. Such effects may also partly explain differences in selectivity between different candidate drugs designed to affect the same pathway/receptor but which present with different pharmacological/side effect profiles. If these effects on the metabotype are directly related to the concentrations of the drug in the circulation, then they ought to be easy to detect, as they should wax and wane in response to a compound's pharmacokinetics (or those of a bioactive drug metabolite). Previously we have described preliminary results that seemed to support this conjecture for a number of medium to non‐polar metabolites present in mouse urine and detected via the reversed‐phase profiling of urine using UHPLC‐IM‐MS [9]. These changes were detected following the IV administration of gefitinib (iressa) a tyrosine kinase inhibitor (TKI) targeting the epidermal growth factor receptor (EGFR‐TK) that is used for the treatment of, e.g., non‐small cell lung cancer [10]. The changes in metabolite profiles we observed involved both increases and decreases in the relative amounts of a number of metabolites in response to gefitinib administration and did indeed seem to provide evidence of a “pharmacometabodynamic” effect (defined as “*the dynamic, time‐related, and reversible changes in metabolic phenotypes resulting from the pharmacological effects of a drug (or other bioactive substance) on the metabolome*” [9]). More recently we have shown clear evidence of similar changes in the plasma lipid profiles of the same mice that correlated closely with the pharmacokinetic profile of the drug in these mice [11]. Having noted these pharmacometabo‐ and pharmacolipidodynamic effects in mice dosed with gefitinib, we have extended our coverage of the mouse urinary metabolome using HILIC with a cyclic ion mobility‐spectrometer‐mass spectrometer combination to profile the changes in the polar metabolic urinary metabotypes of mice following oral (PO) administration of the drug.

## 2 Materials and Methods

### 2.1 Solvents and Chemicals

Reagents and solvents for the analysis of mouse urine included LC/MS grade water (H_2_O) methanol (MeOH), acetonitrile (ACN), ammonium acetate, and 0.1% formic acid (FA) and were purchased from Sigma‐Aldrich (Dorset, UK). Instrument calibration was performed using the “Waters Major Mix IMS/ToF Calibration Kit for IMS” (Waters Corp., Milford, USA). Sodium formate, for instrument calibration, and leucine–enkephalin (used as a lockmass) were also obtained from Sigma‐Aldrich. Standards used for the identification of endogenous metabolites were purchased from a variety of sources, including Merck (Dorset, UK).

### 2.2 Study Conduct

Full details of the study's conduct have been described elsewhere [12]. Briefly, 20 male C57Bl/6JRj mice, 9 weeks of age (20.3–26.5 g), were administered either drug free vehicle or gefitinib (50 mg/kg) at Evotec SAS (Tolouse, France). Prior to dosing, a full ethical management review was undertaken by Evotec, with the study approved by CEPAL (No. APAFIS#04932.02) and followed both national and EU guidelines. Drug administration (5 mg/mL) was as a homogeneous suspension prepared in hydroxypropylmethyl cellulose (HPMC)/polysorbate80/water (0.5%/0.1%/99.4% w/w/v). The mice were subdivided into two groups of 10 to give vehicle (*n* = 10) and gefitinib‐treated (*n* = 10) dose groups. The control and gefitinib dose groups were housed, in groups of five by dose, in metabowls for the collection of urine. An overnight (pre‐dose) urine collection was performed prior to drug administration with subsequent collections for the periods 0–3, 3–8, and 8–24 h post dose. All urine samples were then stored at −80°C until analyzed.

### 2.3 Metabolite Profiling of Endogenous Metabolites in Urine

#### 2.3.1 Sample Preparation

Endogenous metabolites present in urine were profiled using 20 µL aliquots of urine. Each sample was mixed first with 20 µL of LCMS‐grade water followed by 350 µL of LCMS‐grade ACN. The samples were vortexed, left to stand for ca. 10 min (at 2C–8°C), then centrifuged (13 000 rcf, 10 min). A 150 µL aliquot of the supernatant was then taken for analysis, while another aliquot (20 µL) of each sample was pooled to provide a suitable quality control (QC) sample [13]. The study samples were then randomized (in triplicate) for analysis. The QC samples were analyzed both at the beginning and end of the analytical run and after every fifth sample during the course of the analysis.

Prior to the analysis of the study samples, five injections of the QC samples were performed to passivate /condition the UHPLC passivate/condition the UHPLC column/chromatography system in order to ensure analyte retention time (t_R_) stability and also to act as a system suitability test (based on an inspection of pre‐analysis t_R_ stability and factors such as signal intensity, peak shape, etc.,). Following the analysis, the QC data were examined for evidence of run order effects and changes in sensitivity over the course, and only features showing RSDs of less than 30% were taken for further multivariate analysis (see below).

#### 2.3.2 Chromatography

HILIC (hydrophilic interaction liquid chromatography) profiling of urine was carried out according to [14] using an I‐Class ACQUITY PLUS UPLC system (Waters Corp., MA, USA) on a 2.1 × 100 mm, 1.7 µm BEH HILIC ACQUITY UPLC column (Waters Corp.). The mobile phases used were 0.1 % (v/v) FA plus 10 mM ammonium acetate in ACN:H_2_OWater (95:5 v/v) as solvent A and ACN:0.1% (v/v) FA plus 10 mM ammonium acetate mixed (1:1 (v/v) as solvent B. The flow rate was 0.4 mL/min, and the column was maintained at a temperature of 40°C. Separation was via gradient elution starting with 1% B for 1 min, then a linear increase to 100% B over 11 min, at which time the system was returned to the starting conditions (0.1 min) and re‐equilibrated for 4 min between analyses. An injection volume of 3 µL of each diluted urine sample was employed.

#### 2.3.3 Ion Mobility‐Mass Spectrometry

MS data were collected using both positive (+ve) and negative (−ve) electrospray ionization (ESI) using a SELECT SERIES Cyclic ion mobility (cIM‐MS) mass spectrometer (Waters Corp., Wilmslow, UK) [15] operated in single pass, resolution (V optics) mode. The cIMS data were collected in continuum mode using HDMS^E^ over the *m*/*z* range 50–1200 with a scan time of 0.1 s [16, 17]. Low energy (MS1) experiments used a collision energy of 4 eV, whilst elevated energy (MS2) experiments, used to obtain fragment ion data, employed a linear collision energy ramp of 19–45 eV. To ensure mass accuracy leucine enkephalin (leu‐enk, 200 pg/µL) was infused at 20 µL/min via the lockspray interface with data acquired every 30 s. The capillary voltage was set to 1.0 kV for +ve and 2.0 kV for −ve ESI. A cone voltage of 25 V, a source temperature of 120°C and a desolvation gas temperature of 600°C were used with the cone gas (N_2_) flow rate set at 50 L/h and the nebulization gas (N_2_) flow at 800 L/h. Ion mobility (IM) settings were cIM pressure 2.3 mbar (nitrogen) and cIM travelling wave height 35 V, at a constant velocity of 375 m/s. The IM device was calibrated with the Major Mix IMS Calibration Kit (Waters Corp.,) over the collision cross section (CCS) range 130–306 Å^2^ enabling CCS values to be obtained in N_2_. The ToF was calibrated using 0.5 mM sodium formate. Data were collected using a web‐based user interface based on existing MassLynx software (Waters Corp.).

#### 2.3.4 Data Analysis for Metabolite Identification

LC‐MS data were aligned for HILIC retention times (t_R_) and ionization using the pooled QC samples and normalized for *mz*/t_R_ pairs (features) using Progenesis QI (Nonlinear Dynamics, Newcastle upon Tyne, UK) resulting in an aggregate file enabling peak picking and accounting for any missing values. The resulting data sets were then analyzed using EZInfo (Sartorius, Umeå, Sweden) and MetaboAnalyst [18]. Principal component analysis (PCA), with pareto scaling (where the variables were first centered and then multiplied by 1/√*S*_K_ (where *S*_K_ = that variables standard deviation (SD)). PCA was used to assess data quality and investigate dose related changes in the metabolome. To aid correlation analysis/pattern searching hierarchical clustering and Pearson's *R* coefficient were also used. Molecular features highlighted via statistical analysis were initially annotated using accurate mass, isotopic fit, and comparison with MS spectra from reference standard and in silico generated fragmentation mass spectra provided by databases such as the HMDB (Human Metabolite Database, version 5.0) [19] and PubChem (https://pubchem.ncbi.nlm.nih.gov/). The experimental CCS values obtained for these features were then compared with those derived using an in‐house CCS prediction algorithm [20] to reduce the subsequent “search space.” Endogenous metabolites of interest in urine annotated as a result by this process were then compared with data from authentic standards whenever possible (as indicated in text and tables) to provide confirmation of their identities using in‐house databases of authentic standards, based on mass spectral (MS)and precursor and fragmentation data as well as g a HILIC data base for retention time (t_R_) and CCS data (see Tables S1 and 2). Initial peak identification criteria were t_R_ ± 0.5 min, CCS ± 2.5%, and MS 10 (precursor) and 15 ppm (fragments) respectively.

## 3 Results and Discussion

The analysis of polar metabolites is often problematic in metabolic phenotyping studies and to address this challenge, HILIC has been widely employed, providing improved polar analyte retention compared to RPLC with acceptable separation performance [21, 22]. Further, HILIC does not require the use of mobile phase additives such as, e.g., ion pairing agents, which can result in ion suppression in ESI‐MS. While polar analytes, such as organic acids, biogenic amine, oxylipins, and amino acids, etc., are retained with HILIC, lipophilic analytes are not and elute early in the separation. The method used here provided an average analyte peak width of 6 s at the base, giving a peak capacity of ca. 60 for the 6 min separation space. To increase the overall peak capacity, we then employed an IM separation. Since the first demonstration of HPLC‐IM‐MS for metabolic phenotyping [23], the methodology has been increasingly adopted by the field (reviewed in e.g., [24, 25]). The hyphenation of UHPLC with the second, orthogonal, dimension of separation provided by IM is perfectly suited for this type of analysis. As the width of the chromatographic peaks are a few seconds, while the IM separation occurs in milliseconds and the subsequent ToF MS detection only requires microseconds. This allows the IM to sample each LC peak multiple times, and similarly, the IM peak can also be measured by the ToF detector enough times to ensure complete definition of the analyte peak. The addition of IM brings the same sort of advantages to complex sample analysis as 2D LC by enabling the separation of co‐eluting species prior to MS detection. Adding IM also greatly increases the number of t_R_/MS features detected (by more than 50% for short 5–10 min separation [17]) but has the advantage of not increasing analysis time. In addition to increasing feature count, this improved resolution can also have the additional benefit of improving the subsequent MS data, often greatly simplifying metabolite identification. The use of HILIC‐IM‐MS for the metabolic phenotyping of urine has previously been demonstrated using a linear TW IMS device with a path length of 40 cm and a resolving power of approximately 40 [26]. Here we have used HILIC with the cyclic IMS, which offers the advantage over the linear TW cell IMS of a longer ion path of some 95 cm, providing a greater separation space and a resolving power in a single pass of ca. 65 [27] .Resolution of co‐migrating metabolites can be increased if needed by sending ions around the ion path multiple times (see [27]). Indeed, using the multi‐pass option provides a means of increasing the separation path length to up to 30 m to increase resolution to > 400. However, while the use of multiple passes is beneficial for selected parts of the separation, in practice we have found it most useful for “targeting” particular peaks of interest [27]. Employ this approach in routine untargeted “profiling method” can be problematic, as fast‐migrating ions can begin to “lap” the slower ones after several passes, resulting in analyte overlap in the IM cell and incorrect measurement of CCS values. Here we employed the extra resolution provided by a single pass of ions through the cIMS cell compared to that of the linear TW format to enhance the overall separation capability of the HILIC method.

### 3.1 Untargeted Analysis of Urine, Including Gefitinib and Its Metabolites, Following PO Administration

**FIGURE 1:**
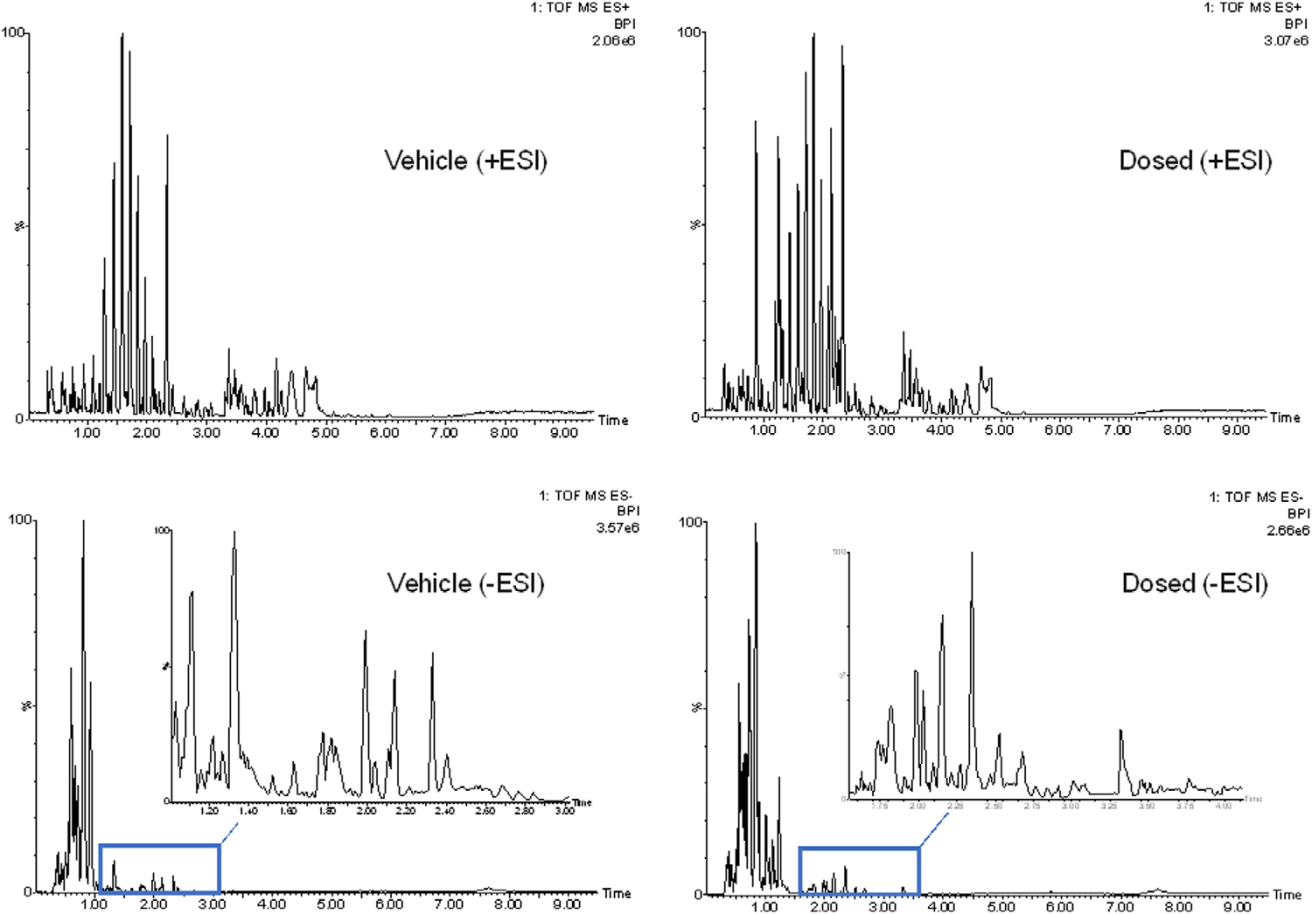
Representative HILIC‐cIM‐MS +ve and −ve ESI‐base peak chromatograms of mouse urine obtained from 3–8 h vehicle (left) and gefitinib‐dosed (right) mouse urine samples. A number of the large peaks eluting in the first 2.5 min for +ve ESI, or 1.0 min for −ve ESI were due to due to methyl‐ and ethyl‐cellulose‐related contaminants (mass spectra in Figure S1).

Following UHPLC‐IM‐MS analysis, examination of the UHPLC‐MS data showed that all of the QC samples were acceptable, with t_R_ variation over the course of the analysis observed to be less than 0.5% for all *m*/*z*/t_R_ pairs and no changes in peak shape or signal intensity observed. Representative chromatograms are provided in Figure 1 for the 3–8 h vehicle and gefitinib dosed groups. On a detailed review of the mass chromatograms obtained for urine however, it was clear that a number of the early eluting peaks (0–2.5 min, that were present in all of the samples, including the pre‐dose urines, were due to contamination with various methyl and ethyl celluloses (see Figure S1). As these peaks were present both pre‐dose and post administration samples from both vehicle (control group) and gefitinib‐dosed mice they clearly did not result from the excipients used to solubilize the drug (i.e., (HPMC)/polysorbate 80). As described in detail elsewhere both gefitinib and its metabolites were also excreted via the urine, with peak concentrations seen in the 3–8 h post dose collection [12]. The impact of gefitinib administration on the polar endogenous metabolite profiles was assessed, following removal of the data for the methyl and ethyl cellulose contaminants as well as that resulting from the presence of gefitinib and its metabolites, on the urine of PO‐dosed mice. For multivariate statistical analysis (MVA) only those features having a CV of < 30% in the QC samples were used which amounted to ca. 10 000 and 5400 ions for data acquired in +ve and −ve ESI modes respectively.

The scores plot for the principal components analysis (PCA) of the +ve ESI data obtained from the HILIC‐cIM‐MS of urine is shown in Figure S2 for vehicle‐ and gefitinib‐dosed mice, respectively. This analysis clearly shows differences in the profiles for the respective groups, with, in the case of the gefitinib‐dosed mice, PCs 1 and 2 representing 58% and 14% of the observed variance in the data, respectively. In the plot for both the vehicle‐ and gefitinib‐dosed mice, the pre‐dose samples can be seen to cluster tightly together with a time‐related progression through the 0–3, 3–8, and 8–24 h samples in a clockwise trajectory for the vehicle‐dosed group and an anticlockwise direction for the gefitinib‐administered animals.

**FIGURE 2:**
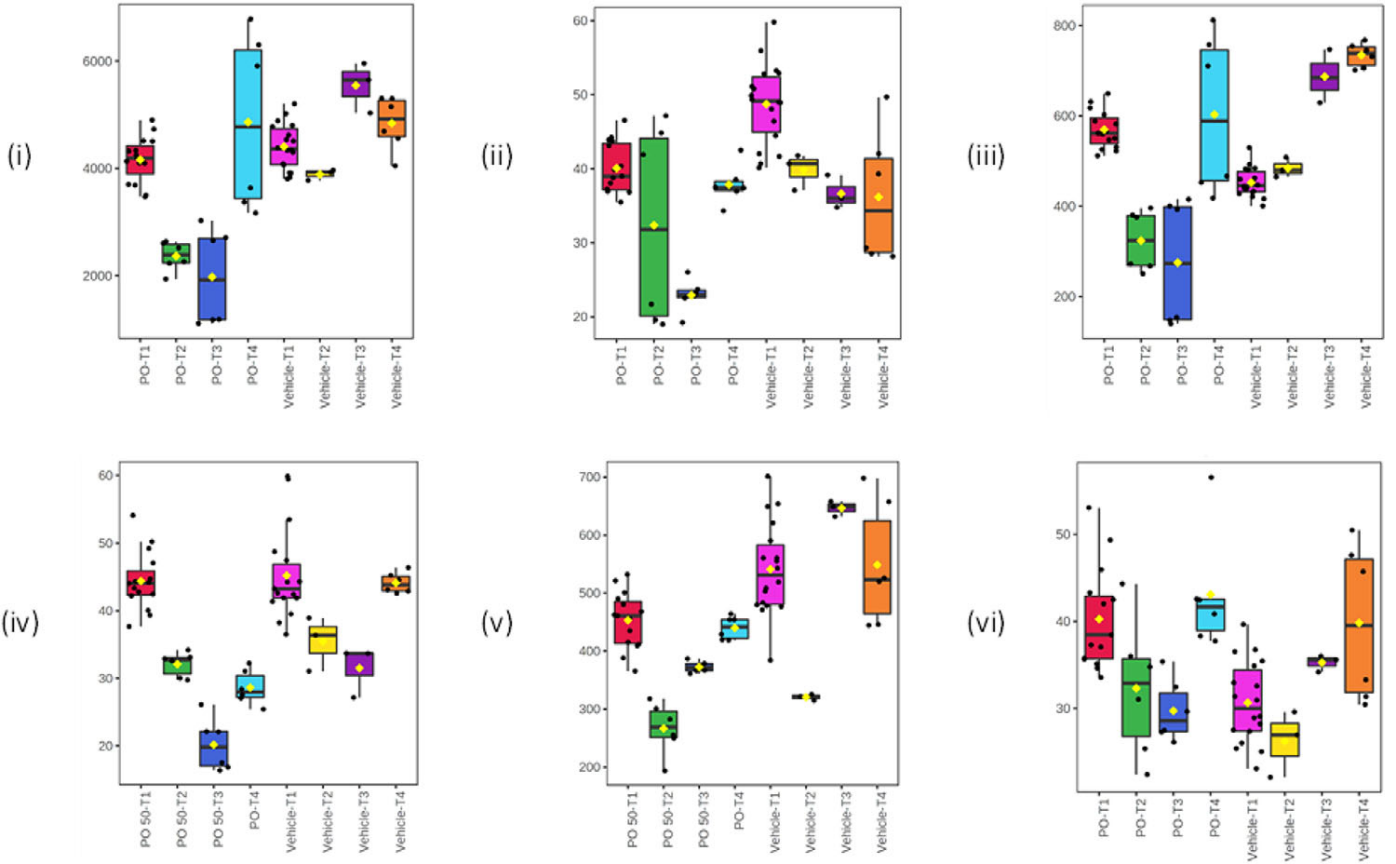
Pharmacometabodynamic variation in the urinary excretion of the endogenous metabolites, i) acetylcarnitine, ii) myristoylglycine, iii) isobutyrylcarnitine, iv) cyclic AMP, v) 3‐phenylpropionylglycine and vi) thymidine. The data for these metabolites were normalized using all features. The four time points are represented as pre‐dose T1 (red‐gefitinib dosed or pink‐vehicle dosed), 0–3 h T2 (green‐gefitinib dosed, yellow‐vehicle dosed), 3–8 h T3 (blue‐gefitinib dosed or purple‐vehicle dosed), and 8–24 h T4 (light blue‐dosed or orange (vehicle). Mass spectra provided in Figure 3 (isobutyrylcarnitine) and Figures S4–S8.

MVA of the PO gefitinib‐dosed group showed significantly greater separation of the 3–8 h sample signals from the pre‐dose group than that observed following vehicle‐only administration (Figure S2). As drug concentrations in the plasma and urine declined (see [12]), the “trajectory” for these +ve ESI data began to return towards the pre‐dose group, as indicated by the position of the 8–24 h sample ellipse. PCA of the vehicle only group showed that PC1 and PC2 accounted for 52% and 14% of the variance observed in these data, respectively, and a clear time‐related trajectory from pre‐dose through the 0–3, 3–8, and 8–24 h urines. Some of the effects noted for the urinary profiles of both vehicle‐ and gefitinib‐dosed mice likely resulted from normal diurnal changes in polar endogenous metabolites (e.g., feeding etc.), and others were possibly due to the excretion of the excipients used for the vehicle. In addition, cage effects showing differences in the position of groups of samples can be seen in the ellipses included in the PCA plots for both +ve and −ve ESI data, depending upon which of the two metabowls the samples (for both drug‐ and vehicle‐dosed mice) had been collected from the various collection periods. Such cage‐dependent differences in metabolic signatures are not uncommon and probably reflect environmental influences rather than drug effects. A similar result was obtained for these urine samples when the −ve ESI data were subjected to PCA (Figure S3).

Heatmaps based on the top 100 metabolic features (based on ANOVA *t*‐tests) responsible for the differences observed between the urines obtained from vehicle‐ and gefitinib‐dosed mice for the +ve ESI and −ve ESI data are provided in Figures S2 and S3, respectively, illustrating the high degree of similarity seen for all of the pre‐dose samples. As can be seen from the heatmaps, the administration of a single 50 mg/kg PO dose of gefitinib resulted in a significant deviation from the pre‐dose samples, consistent with the PCA analysis. The largest effects were most evident at 0–3 h after drug administration, with the 3–8 h post‐dose samples and 8–24 h samples also showing some differences compared to their pre‐dose urinary phenotypes. The 0–3 h post‐dose samples also showed, as indicated above, evidence for cage effects, with the two samples clearly separated in the heatmaps (Figures S2 and 3). These cage‐related differences were also observed in the 3–8 and 8–24 h samples but to a lesser extent. Both +ve and −ve ESI data indicated that the oral administration of gefitinib at a “pharmacological” (subtoxic) dose had resulted in significant changes in the endogenous metabolites eliminated in the urine.

### 3.2 Endogenous Metabolite Identification

**FIGURE 3:**
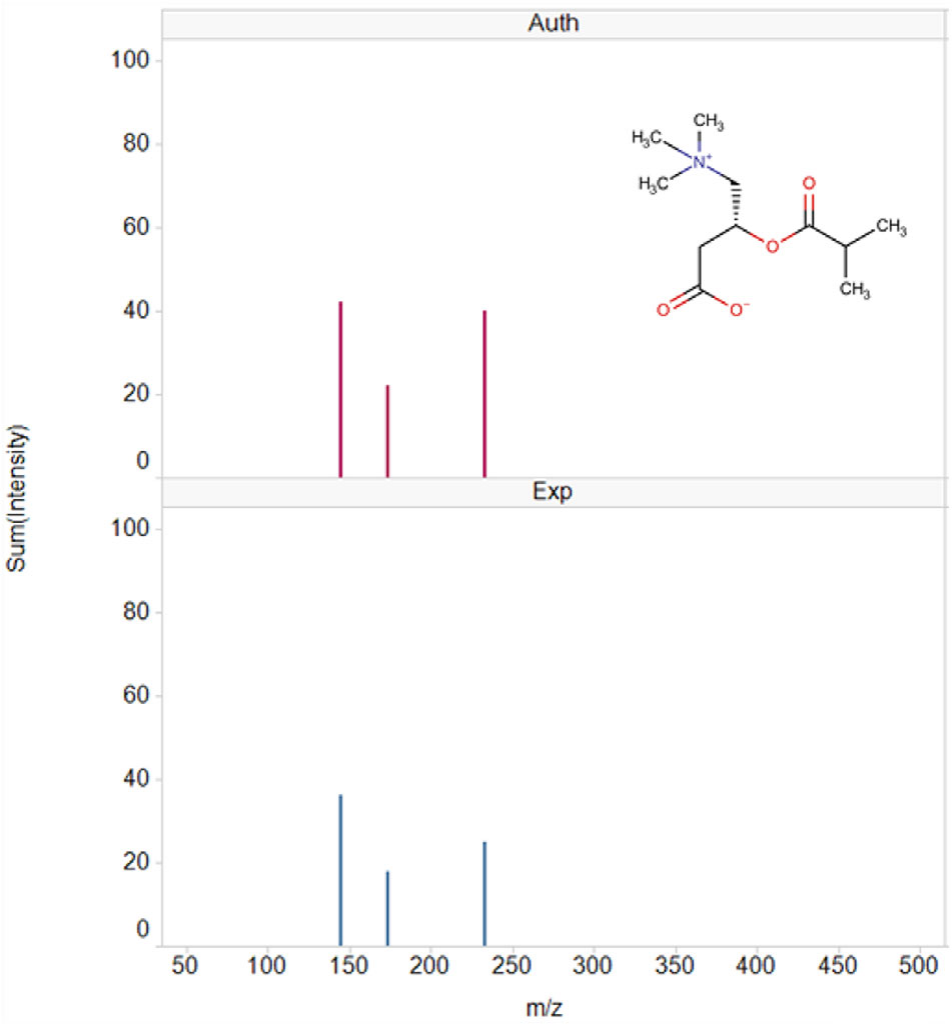
MS/MS spectra for isobutylcarnitine comparing experimentally (Exp) derived data against its authentic standard (Auth). Spectra corresponding with the authentic standard (upper spectrum; red peaks) and Progenesis QI identified urine metabolite (lower spectrum; blue peaks) are shown. The values obtained for the precursor and fragment ions of the experimental and authentic standard are represented as ppm in the inset.

The identification of the endogenous metabolites subject to change following drug administration clearly holds the potential to provide insight into the effects, both intended and unexpected (or indeed unwanted), of EGFR inhibition. However, it is currently the case that unequivocal metabolite identification still represents, a major bottleneck in metabolic phenotyping. Whilst a level of metabolite annotation, of variable quality, can be performed using online databases such as e.g., the HMDB [20], LIPID MAPS [28], LipidBlast [29], and ChemSpider [30], etc., providing a valuable first step, these databases only highlight potential targets. For confirmation of identity, to e.g., Level 1 of the MSI [31], for further investigation, care must be taken not to automatically accept the first “hit,” or even that with the highest matching score, uncritically [32]. Indeed, given the profligacy of biology, it is often the case that multiple compounds with the same nominal mass (and isomeric compounds with the same exact mass) are suggested as potential “biomarkers”. It is also often the case that the lists produced on the basis of such database searches are, on curation, found to be populated with a large number of implausible candidates. These can include secondary plant metabolites, industrial chemicals, pesticides, and pharmaceuticals, etc. These xenobiotics often have no place in laboratory animal‐derived samples and if, they are accepted as being present in the sample, their inclusion requires evidence‐based justification. However, the removal of the obviously unlikely “annotations” does not mean that the remaining “hits” are correct, and final confirmation of identity to the MSI Level 1 [32, 33] is essential in order to be able to develop plausible hypotheses regarding, e.g., pharmacological targets or mechanisms of toxicity [32]. Although MS can often provide compelling evidence for the identity of a metabolite via, e.g., accurate mass measurements, characteristic fragment ions, isotopic patterns, etc., unequivocal identification generally requires a comparison with authentic standards and t_R_ comparisons, etc. Here, in‐house databases of standards, run on the same HILIC system in +ve and −ve ESI, was compared against statistically significant features with precursor accurate mass, characteristic fragment ion patterns, t_R,_ (Progenesis QI) and IM‐derived‐CCS values (see Tables S1 and S2). These data were used to provide metabolite identities at MSI Level 1 [31] wherever possible and resulted in the identification of seven metabolites (Table 1) from the 200 compounds highlighted from +ve and −ve ESI datasets by MVA. A further 3 were characterized as MSI Level 2 (Table 1) based on MS data alone. The mass spectra obtained for two of these metabolites are provided as Figures 3 and 5 with the rest shown in Figures S4–S11. In order to rationalize the changes in the various endogenous metabolites, we have attempted to correlate plasma gefitinib concentrations with changes in endogenous metabolites.

**TABLE 1:** Compounds identified to MSI Levels 1 and 2 from the top 200 *mz*/t_R_ features highlighted as being altered by PO gefitinib administration using MVA.

Compound	Database identifier	ESI polarity	MSI level	Experimental *m*/*z*	Mass error (ppm)	t_R_ (min) (standard)	Adduct	Measured CCS (Å^2^) (*database Std; **calculated CCS)
8‐Hydroxydeoxy‐guanosine	HMDB0003333	+ve	1	284.0978	−3.87	1.94 (2.03)	M+H	154.7 (156.2)*
Thymidine	HMDB0000273	+ve	1	243.0996	8.63	1.35 (1.32)	M+H	144.1 (143.4)*
Myristoylglycine	HMDB0013250	+ve	1	286.2372	−1.40	1.98 (1.98)	M+H	166.0 (167.9)*
Acetylcarnitine	HMDB0000201	+ve	1	204.1237	−1.40	4.18 (4.13)	M+H	137.0 (136.8)*
Isobutyrylcarnitine	HMDB0000736	+ve	1	233.1624	0.86	3.74 (3.70)	M+H	148.5 (150.2)*
Lysyl‐phenylalanine	HMDB0028958	+ve	2	311.2067	−3.53	2.56	M+NH_4_	170.2 (173.0)**
19‐oxotestosterone	HMDB0003959	+ve	2	341.1487	−7.62	1.78	M+K	170.0 (172.7)**
Cyclic AMP	HMDB0000058	−ve	1	328.0451	−0.30	3.31 (3.36)	M−H	164.8 (167.0)*
3‐Phenylpropionyl‐ glycine	HMDB0002042	−ve	1	206.0822	−0.48	1.75 (1.68)	M−H	148.0 (149.7)*
3′‐Asparaginyl‐AMP	CSID58163438	−ve	2	496.0754	−4.43	2.53	M+Cl	204.1 (201.9)**

As already alluded to, such drug‐related “pharmacometabodynamic” [9] and, indeed, pharmacolipidodynamic [11] effects, have the potential to provide valuable insight into the mechanism‐based changes (both on and off target) occurring following drug administration. For 6 of the metabolites highlighted in Table 1, the effect of gefitinib administration was to cause an initial, rapid fall in relative concentrations in the urine of dosed animals compared to the controls (Figure 2i–vi). Thus thymidine, myristoylglycine, acetylcarnitine (mass spectra shown in Figures S4–S6), and isobutyrylcarnitine (Figure 3), detected in +ve ESI, and cAMP and 3‐phenylpropionylglycine, detected using −ve ESI (Figures S7 and S8), all showed a relative reduction in urinary concentrations compared to pre‐dose and post‐dose control values. Maximum depression of the amounts of these metabolites was generally seen in the 0–3 and 3–8 h samples, with a return to control values in the 8–24 h urines.

Myristoylglycine, acylcarnitine, cAMP, 3‐phenylpropionylglycine isobutyrylcarnitine (mass spectrum shown in Figure 3), and thymidine also showed a slight relative decrease compared to their pre‐dose concentrations in the urine of vehicle‐dosed animals, but to a lesser extent than those receiving the drug. By 24 h the relative concentrations of all of these metabolites were similar in both the vehicle and gefitinib dose groups.

**FIGURE 4:**
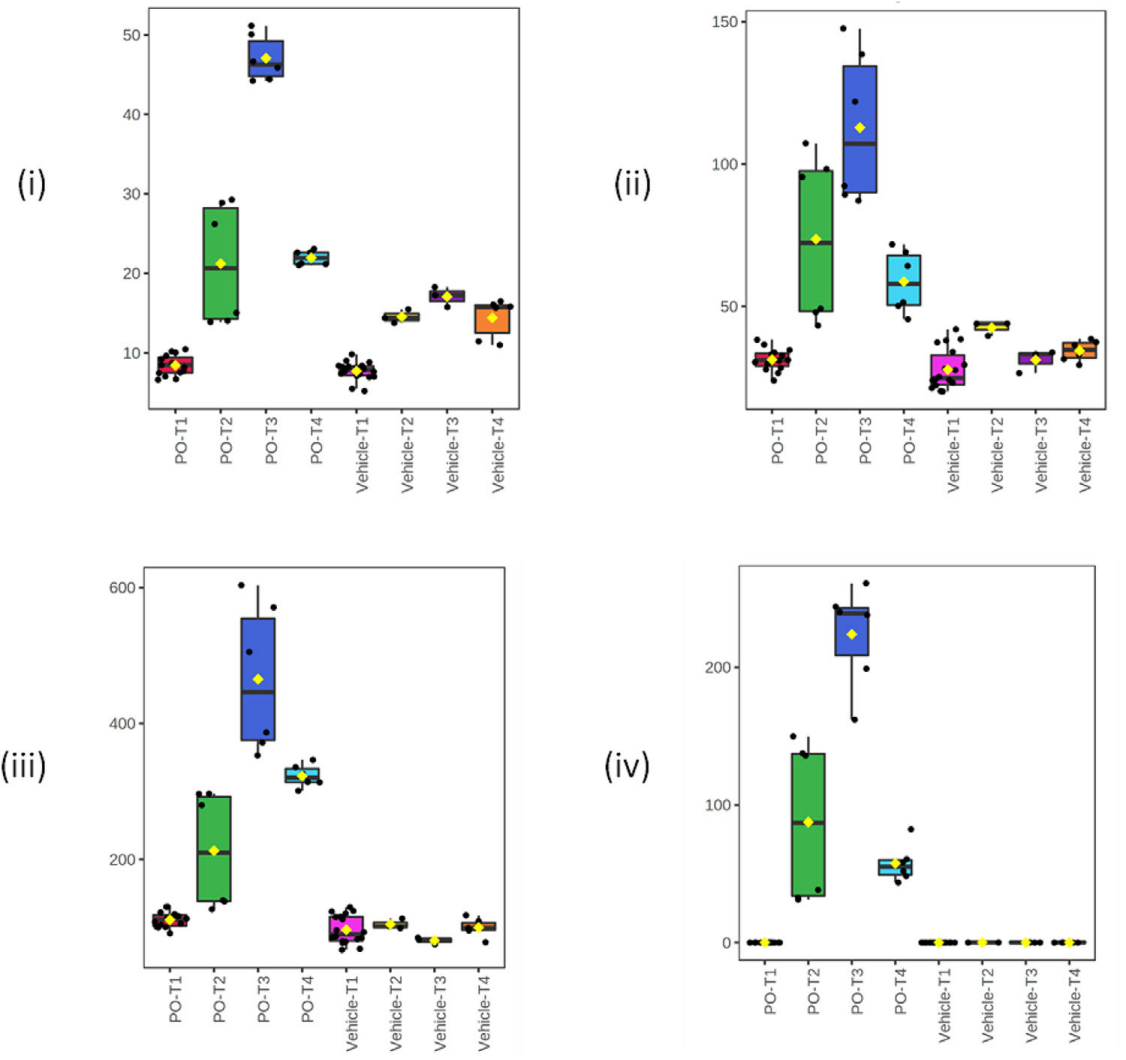
Pharmacometabodynamic variation in the urinary excretion of the endogenous metabolites i) lysylphenylalanine, ii) 19‐oxotestosterone, iii) 8‐hydroxydeoxyguanosine and iv) 3′‐asparaginyl‐AMP. The data for these metabolites were normalized using all features. Key pre‐dose: T1 (red‐dosed or pink‐vehicle)); 0–3 h T2(green‐dosed or yellow‐vehicle); 3–8 h T3 (blue‐dosed or purple‐vehicle); 8–24 h T4(light blue‐dosed or orange‐vehicle). Corresponding MS spectra provided in Figures 5 and S9–S11.

**FIGURE 5:**
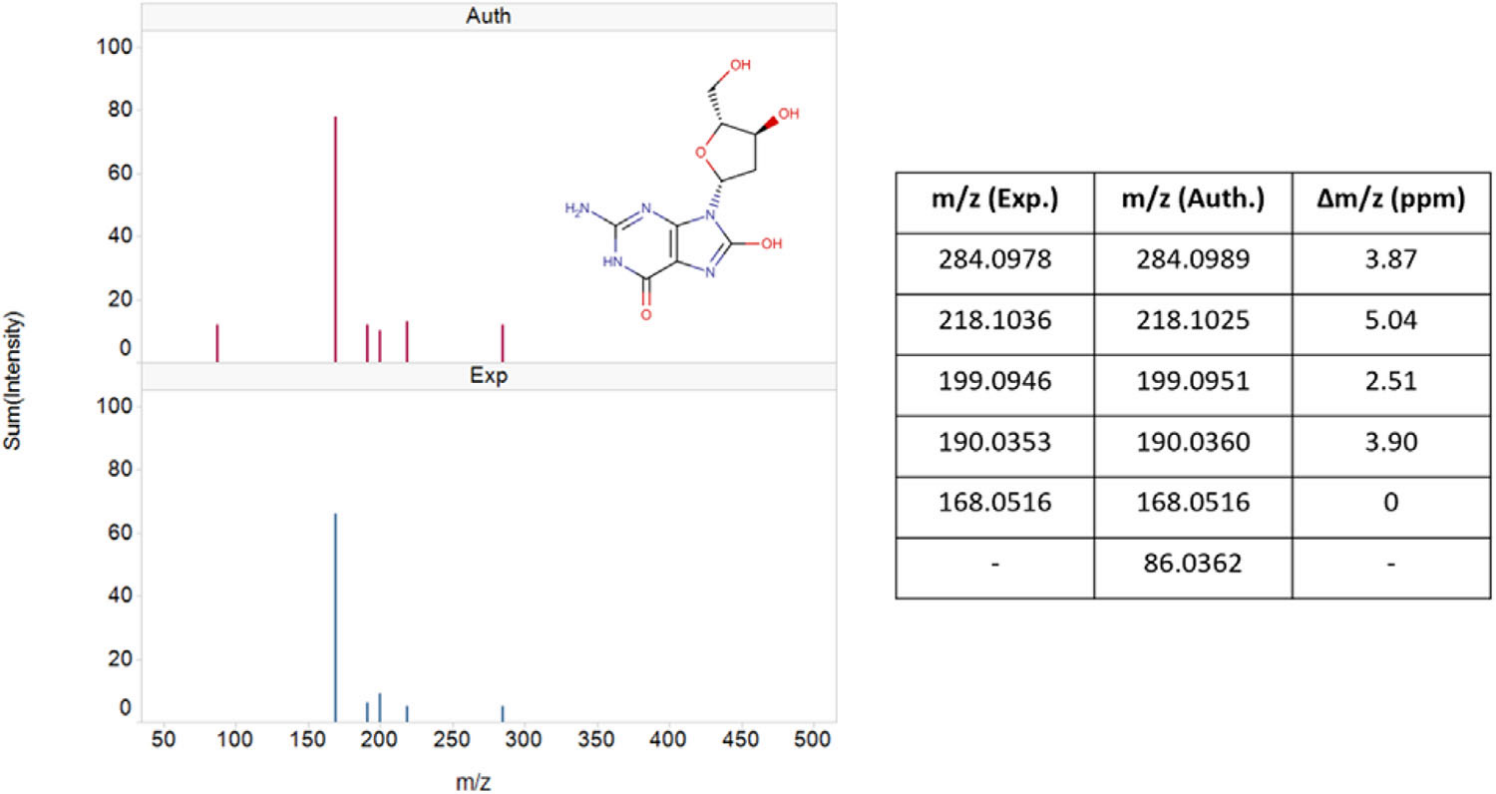
MS/MS spectra for 8‐hydroxydeoxyguanidine comparing experimentally (Exp) derived data against its authentic standard (Auth). Spectra corresponding with the authentic standard (upper spectrum; red peaks) and Progenesis QI identified urine metabolite (lower spectrum; blue peaks) are shown. The values obtained for the precursor and fragment ions of the experimental and authentic standard are represented as ppm in the inset.

In contrast to these time‐dependent decreases, the metabolites lysylphenylalanine, 8‐hydroxy‐deoxyguanosine, and the peaks annotated as potentially being 19‐oxotestosterone and 3′‐asparaginyl‐AMP showed relative increases in concentration when compared to the vehicle group following dosing (Figure 4). The product ion mass spectrum for 8‐hydroxy‐deoxyguanosine is shown in Figure 5, while the MS data for lysylphenylalanine, 19‐oxotestosterone, and 3′‐asparaginyl‐AMP are provided in Figures S9–S11, respectively.

Inspection of the excretion profiles showed that, for all of these metabolites, there were significant increases in relative concentrations following the administration of gefitinib, while concentrations in samples from the vehicle‐only mice generally remained steady.

Many other features showed similar behaviors to the metabolites discussed above, but their identities remain to be confirmed. Clearly, the 10 metabolites identified here represent a relatively small proportion of the total number of compounds detected as having been changed but, as is well known, confident metabolite identification remains a significant bottleneck in metabolomic investigations. Attempts to improve this total via e.g., the use of in silico MS prediction tools did not inspire confidence and were not explored further.

## 4 Discussion

As described elsewhere, following PO administration at 50 mg/kg the plasma pharmacokinetics of gefitinib provided peak observed plasma concentrations of ca. 7 µg/mL in mice 1 h post dose, declining rapidly thereafter with a T_1/2_ of 2.6 h, with significant amounts of the drug and its metabolites excreted in the urine [12]. Previously we have shown, using RP‐UHPLC‐IM‐MS, that intravenous (IV) administration of gefitinib to mice was associated with drug‐related changes to the urinary phenotypes of mice that correlated with drug exposure [9]. Here, HILIC‐based UHPLC‐cIM‐HRMS profiling of the polar urinary phenotypes and subsequent multivariate statistical analysis also identified numerous changes over the 24 h period following PO administration of gefitinib at 50 mg/kg. Similar analysis of the existing pharmacokinetic data [12] with the endogenous metabolic phenotyping data have also highlighted effects (presumably a combination of both on‐ and off‐target) of the drug on the polar metabolites present in the urine of these PO‐dosed mice. Thus, compared to the vehicle‐dosed control mice, the polar urinary metabolite profiles of PO gefitinib‐dosed animals showed significant changes for endogenous metabolites (both in increased and decreased relative concentrations) over the period 24 h post drug administration. Many of these changes, whether relative increases or decreases, coincided with the period covering the maximal circulating plasma concentrations of gefitinib. Metabolite profiles then generally showed a return towards control values by 24 h post‐dose, by which time the drug, and its metabolites, had largely been eliminated from the systemic circulation [12]. As noted above, six endogenous metabolites (MSI Level 1)—acylcarnitine, isobutyrylcarnitine, myristoylglycine, 3‐phenylpropionylglycine, cyclic AMP, and thymidine—showed relative decreases in concentration compared to the vehicle‐only controls. A further four metabolites, 8‐hydroxydeoxyguanosine (MSI Level 1) and lysylphenylalanine, 19‐oxotestosterone, and 3′‐ asparaginyl‐AMP, (all MSI Level 2) showed relative increases. The significance of these changes is difficult to assess in the absence of flux experiments, as such changes in the amounts of metabolites detected may reflect either the increased/decreased biosynthesis or consumption of a particular metabolite. However, taken simply as an indication of a change in metabolism, these point to pathways involving, e.g., DNA biochemistry. If so, it can be conjectured that oxidative damage and repair are indicated by elevated 8‐hydroxydeoxyguanosine, which is also a known biomarker of, e.g., cancer or inflammation. Further indications of DNA repair may be provided by decreased amounts of thymidine [34, 35, 36].

The changes seen for acylcarnitine, isobutyrylcarnitine, 3‐phenylpropionylglycine, and myristoylglycine may indicate effects on energy production and lipid metabolism via beta‐oxidation. In addition, changes in cyclic AMP (cAMP) may also reflect effects on energy production/lipid metabolism. However, given the range of its roles in, e.g., acting as a second messenger with effects, e.g., gene expression, cell growth/differentiation, metabolism, etc., this is clearly speculation. The various changes in metabolic processes identified here do, however, suggest potential further lines of research using, e.g., in vitro systems and flux experiments, etc., to further characterize the pharmacological properties of gefitinib.

The potential roles of the remaining three metabolites (lysylphenylalanine, 19‐oxotestosterone, and 3′‐asparaginyl‐AMP), which were only annotated to MSI Level 2, will not be speculated on except to say that these peaks provide examples of compounds that showed a very clear initial relative increase in concentration following oral administration of gefitinib, followed by a decrease that mirrored the plasma concentration profile seen in plasma [12], and that further investigation, including definitive identification, is clearly warranted.

The temporal behavior shown by the metabolites and many other unidentified compounds highlighted here strongly supports a time‐ and concentration‐linked response. We have, by analogy to pharmacodynamic responses, previously termed this pharmacometabodynamics and defined it as “the dynamic, time‐related, and reversible changes in metabolic phenotypes resulting from the pharmacological effects of a drug (or bioactive substance) on the metabolome” [9]. However, our ability to further exploit this phenomenon to better understand the pharmacology of gefitinib and other compounds in its class is currently limited by our inability to rapidly and confidently, identify the many endogenous metabolites seen to be affected by the drug. Thus, the characterization and unequivocal identification of more of these metabolites, which is essential for biochemical interpretation, remains a major limitation of current untargeted metabolic phenotyping studies. The construction of in‐house databases of authentic standards can clearly help with the identification, and also the exclusion, of metabolites from consideration, but requires the standards to be readily available. Thus our internal HILIC‐IM‐MS database of endogenous metabolites (Tables S1 and S2) enabled ca. 200 common endogenous metabolites to be excluded from consideration. Public databases can be of some assistance in highlighting potential structures, but when the MS data provided are not based on authentic standards, they are of rather limited value. Emerging programs for predicting MS data show some promise but, in our experience, are fallible. This opinion is based on the observation that when used to predict the mass spectra for endogenous metabolites for which we had data from authentic standards, they provided similar spectra in only ca. 50% of the (limited number) of cases that we tested them on. No doubt such predictive software will improve with time, but for the moment, their use with caution seems advisable.

These pharmacometabodynamic data were obtained from samples obtained in a study designed to investigate the pharmacokinetics of gefitinib and determine its metabolites in the mouse, including in tissues and excreta [12]. The metabolic phenotyping data reported here thus represents an approach to extract additional, potentially valuable, information from this study concerning the effects of gefitinib on the biochemistry of these mice. Such use of samples obtained initially for other purposes thereby maximizes information recovery from the animal studies and thus may limit the need for further in vivo studies while providing biochemical insights into MOAs and suggesting candidate endogenous metabolites as potential translational biomarkers. Such an approach, greatly facilitated by recent developments in analytical technology, could be readily implemented into modern DMPK studies in early drug discovery and is consistent with the principles of the reduce, replace, refine (3Rs) philosophy for animal experimentation [37]. However, whilst such studies can be used to provide an indication of the wider effects of bioactive substances such as drugs, the approach is clearly subject to a number of limitations. In particular, animal welfare considerations meant that these mice were housed in groups of five, rather than individually. Whilst such a study design does not impact sampling blood from individual animals, it does result in pooled urine samples where interindividual animal variation is lost. Notwithstanding this limitation, results of this sort, obtained in drug discovery, could clearly be used to guide further research to aid drug development. In addition, this approach, with its very modest sample requirements, could easily be translated into later studies, including those in patients. Obviously, if the results of these modest studies were deemed sufficiently important to require validation, a further study could easily be designed to confirm that the apparent pharmacometabodynamic effects were real and further explore and validate them.

## 5 Conclusions

Urine analysis by HILIC‐cIM‐MS showed clear differences in the polar metabolome of gefitinib versus vehicle‐dosed mice. These changes in metabolic phenotypes mirrored the plasma and urinary concentration versus time profiles of the drug and its metabolites, suggesting that they were “pharmacometabodynamic” effects that were driven by the pharmacology of gefitinib. Results such as these, while clearly limited by both the small number of animals employed and the current low rate of unequivocal endogenous metabolite identification, highlight the potential for metabolic phenotyping to detect the time‐related effects of drugs (and other bioactive species) on the metabolome, which may lead to a better understanding of their pharmacological and toxicological effects.

## Author Contributions

Conceptualization: Robert S. Plumb and Ian D. Wilson. Methodology: Adam King and Lee A. Gethings. Writing — original draft preparation: Ian D. Wilson, Lee A. Gethings, Robert S. Plumb. All authors have done writing, review and editing.

## Conflicts of Interest

IDW has previously acted as a consultant to a number of companies including Waters Corp., the other authors declare no conflicts of interest.

## Supporting information



Supporting Information

## Data Availability

The authors confirm that the data supporting the findings of this study are available within the article and its supporting information.
