# Supplementary material for: A HILIC‐IM‐MS‐Based Pharmacometabodynamic Study of the Effects of Orally Administered Gefitinib on the Polar Urinary Metabolic Phenotypes of C57Bl6 Mice
Source: J Sep Sci. 2025 May 6;48(5):e70163. doi: 10.1002/jssc.70163 (PMC12053960; doi:10.1002/jssc.70163)
Supplement: Supplementary file 1 — Supporting Information [file JSSC-48-e70163-s001.docx]

**SUPPLEMENTARY MATERIAL**

**A HILIC-IM-MS-Based Pharmacometabodynamic Study of the Effects of Orally Administered Gefitinib on the Polar Urinary Metabolic Phenotypes of C57Bl6 Mice**

Adam King^1^, Lee A. Gethings^1^, Robert S. Plumb^2^, Ian D. Wilson^3*^

^1^ Waters Corporation, Stamford Avenue, Wilmslow, SK9 4AX, UK ^2^ Waters Corporation, Milford, MA, 01757, USA. ^3^ Division of Systems Medicine, Department of Metabolism, Digestion and Reproduction, Imperial College London, UK.

**Contents**

**3: Table S1:** Measured authentic standards (+ve ESI) used to generate an in-house database for searching via Progenesis QI.

**9: Table S2:** Measured authentic standards (-ve ESI) used to generate an in-house database for searching via Progenesis QI.

**18: Figure S1:** Characteristic fragmentation spectra representing methyl and ethyl cellulose (mass error = 0.44 ppm)

**19: Figure S2:** Unsupervised principal component analysis (PCA) and associated heatmap (+ ve ESI) relating to the vehicles and oral dosed groups. The PCA analysis includes all features adhering to a CV filtering of <30%, whilst the heatmaps correspond to the top 100 features based on ANOVA t-test. Heatmaps were constructed using Euclidean distance and Ward clustering.

**20: Figure S3:** Unsupervised principal component analysis (PCA) and associated heatmap (-ve ESI) relating to the vehicles and oral dosed groups. The PCA analysis includes all features adhering to a CV filtering of <30%, whilst the heatmaps correspond to the top 100 features based on ANOVA t-test. Heatmaps were constructed using Euclidean distance and Ward clustering.

**21: Figure S4:** MS/MS spectra for thymidine comparing experimentally (Exp) derived data against its authentic standard (Auth). Spectra corresponding with the authentic standard (upper spectrum; red peaks) and Progenesis QI identified urine metabolite (lower spectrum; blue peaks) are shown. The difference in mass observed between the precursor and fragment ions of the experimental and authentic standard are represented as ppm in the associated table.

**22: Figure S5:** MS/MS spectra for myristoylglycine comparing experimentally (Exp) derived data against its authentic standard (Auth). Spectra corresponding with the authentic standard (upper spectrum; red peaks) and Progenesis QI identified urine metabolite (lower spectrum; blue peaks) are shown. The difference in mass observed between the precursor and fragment ions of the experimental and authentic standard are represented as ppm in the associated table.

**23: Figure S6:** MS/MS spectra for acetylcarnitine comparing experimentally (Exp) derived data against its authentic standard (Auth). Spectra corresponding with the authentic standard (upper spectrum; red peaks) and Progenesis QI identified urine metabolite (lower spectrum; blue peaks) are shown. The difference in mass observed between the precursor and fragment ions of the experimental and authentic standard are represented as ppm in the associated table.

**24: Figure S7:** MS/MS spectra for cAMP comparing experimentally (Exp) derived data against its authentic standard (Auth). Spectra corresponding with the authentic standard (upper spectrum; red peaks) and Progenesis QI identified urine metabolite (lower spectrum; blue peaks) are shown. The difference in mass observed between the precursor and fragment ions of the experimental and authentic standard are represented as ppm in the associated table.

**25: Figure S8:** MS/MS spectra for 3-phenylpropionylglycine comparing experimentally (Exp) derived data against its authentic standard (Auth). Spectra corresponding with the authentic standard (upper spectrum; red peaks) and Progenesis QI identified urine metabolite (lower spectrum; blue peaks) are shown. The difference in mass observed between the precursor and fragment ions of the experimental and authentic standard are represented as ppm in the associated table.

**26: Figure S9:** MS/MS spectra for lysylphenylalanine based on experimental data only (i.e., authentic standard commercially unavailable).

**27: Figure S10:** MS/MS spectra for 19-oxotestosterone based on experimental data only (i.e., authentic standard unavailable

**28: Figure S11:** MS/MS spectra for 3’-L-asparaginyl-AMP based on experimental data only (i.e., authentic standard commercially unavailable).

**Table S1:** Measured authentic standards (+ve ESI) used to generate an in-house database for searching via Progenesis QI.

| **Name** | **Retention Time (min)** | **CCS (angstrom^2)** | **Adduct** |
| --- | --- | --- | --- |
| Guanosine | 3.8 | 169.37 | M+Na |
| Guanosine | 3.8 | 157.43 | M+H |
| Guanosine | 3.8 | 169.48 | M+K |
| Phenylpropionylglycine | 1.24 | 142.82 | M+H |
| D-Trehalose dihydrate | 5.74 | 175.82 | M+K |
| D-Trehalose dihydrate | 5.74 | 171.81 | M+Na |
| D-fructose | 4.36 | 135.56 | M+Na |
| 4-Aminohippuric Acid | 2.72 | 153.12 | M+H |
| 3-Hydroxytryptamine | 2.06 | 135.93 | M+H |
| 3-Beta-hydroxy-5-alpha-pregnan-20-one | 0.42 | 178.52 | M+H |
| Nicotinuric acid | 3.75 | 137.15 | M+H |
| 3-Methoxytyramine HCl | 0.48 | 134.96 | M+K |
| DL-Arginine | 6.03 | 133.66 | M+H |
| 3-Hydroxyanthranilic acid | 1.16 | 130.93 | M+H |
| Homoarginine hydrochloride | 5.98 | 137.71 | M+H |
| Thyroxine | 2.22 | 215.38 | M+H |
| 2,4,5-Trimethoxycinnamic acid | 0.47 | 151.62 | M+H |
| Xanthine | 4.06 | 132.67 | M+Na |
| Xanthine | 4.06 | 123.82 | M+H |
| *N*-acetyl-L-glutamine | 4.36 | 140.15 | M+Na |
| Cystine | 6.25 | 145.24 | M+H |
| Naringenin | 0.46 | 158.9 | M+H |
| 5-Alpha-dihydrotestosterone | 0.41 | 169.72 | M+H |
| 5-Alpha-dihydrotestosterone | 0.41 | 174.6 | M+Na |
| Phenylacetylglutamine | 2.95 | 163.72 | M+K |
| Phenylacetylglutamine | 2.95 | 163.08 | M+Na |
| Phenylacetylglutamine | 2.95 | 156.68 | M+H |
| Phytonadione | 0.4 | 220.38 | M+H |
| Chlorogenic acid | 3.64 | 192.81 | M+Na |
| Chlorogenic acid | 3.64 | 194.43 | M+K |
| 7-Methylguanine | 2.04 | 136.5 | M+Na |
| Adenine | 1.59 | 119.58 | M+H |
| *N-*Acetyl-neuraminic acid | 5.76 | 167.76 | M+K |
| *N*-Acetyl-neuraminic acid | 5.76 | 165.28 | M+Na |
| *N*-Acetyl-neuraminic acid | 5.76 | 163.58 | M+H |
| Lithocholic acid | 0.41 | 195.28 | M+Na |
| Carnosine | 6.07 | 147.44 | M+H |
| 5-Methyl-2-deoxycytidine | 2.33 | 160.19 | M+K |
| 5-Methyl-2-deoxycytidine | 2.33 | 156.75 | M+Na |
| *N*-methyl-L-phenylalanine | 2.99 | 135.34 | M+H |
| Urocanic acid | 1.68 | 125.91 | M+H |
| 3-(4-Hydroxyphenyl)-propionic Acid | 0.43 | 127.66 | M+H |
| *N*-4-acetylcytidine | 1.93 | 166.95 | M+K |
| *N*-4-acetylcytidine | 1.93 | 166.28 | M+Na |
| Testosterone | 0.44 | 168.85 | M+H |
| Inosine-5-monophosphate (sodium salt hydrate) | 5.98 | 166.14 | M+Na |
| Inosine-5-monophosphate (sodium salt hydrate) | 5.98 | 169.38 | M+H |
| gamma-Glutamylcysteine | 5.91 | 154.6 | M+H |
| gamma-Glutamylcysteine | 5.91 | 153.06 | M+Na |
| methyl 6-hydroxynicotinate | 1.9 | 124.58 | M+H |
| Guanine | 3.78 | 130.77 | M+Na |
| Guanine | 3.78 | 123.98 | M+H |
| Glycyl-L-tyrosine | 5 | 153.59 | M+H |
| Indole-3-butyric acid | 0.46 | 140.16 | M+H |
| 2-deoxyadenosine | 1.27 | 156.8 | M+Na |
| 2-deoxyadenosine | 1.27 | 149.14 | M+H |
| Xanthosine-dihydrate | 4.07 | 166.74 | M+K |
| Xanthosine-dihydrate | 4.07 | 167.2 | M+Na |
| Xxanthosine-dihydrate | 4.07 | 155.62 | M+H |
| Adenosine-monophosphate | 5.84 | 166 | M+H |
| Adenosine-monophosphate | 5.84 | 173.68 | M+Na |
| Maltose | 5.45 | 177.63 | M+K |
| Maltose | 5.45 | 173.5 | M+Na |
| 4-Ethoxyphenylacetic acid | 0.43 | 138.74 | M+Na |
| D-mannose | 4.36 | 135.56 | M+Na |
| Pyridoxal-phosphate | 4.46 | 146.53 | M+H |
| Taurocholic acid | 3.01 | 209.86 | M+Na |
| Taurocholic acid | 3.01 | 213.5 | M+K |
| Taurocholic acid | 3.01 | 207.03 | M+H |
| Indole-5-carboxylic acid | 2.78 | 125.76 | M+H |
| 3-Indolepropionic acid | 1.98 | 136.63 | M+H |
| D-Glucosamine-hydrochloride | 2.59 | 130.06 | M+H |
| DL-normetanephrine | 0.86 | 140.12 | M+Na |
| NADH | 6.14 | 233.08 | M+H |
| 3-Methylxanthine | 1.3 | 126.06 | M+H |
| D-galactose | 4.36 | 135.56 | M+Na |
| Adenosine-5-triphosphate | 6.64 | 193.85 | M+H |
| 5-Methylcytosine | 2.19 | 117.6 | M+H |
| Kynurenic acid | 2.77 | 132.24 | M+H |
| 3-Chloro-l-tyrosine | 3.94 | 147.23 | M+H |
| Progesterone | 0.38 | 176.56 | M+H |
| 2-Deoxyguanosine-5-monophosphate | 6.02 | 166.32 | M+Na |
| 2-Deoxyguanosine-5-monophosphate | 6.02 | 171.32 | M+H |
| Glycochenodeoxycholic acid | 1.69 | 204.08 | M+K |
| Glycochenodeoxycholic acid | 1.69 | 200.53 | M+Na |
| gamma-Glutamylalanine | 5.93 | 147.25 | M+H |
| Caffeine | 0.47 | 134.86 | M+H |
| *N*-Acetyl-DL-tryptophan | 1.57 | 152.84 | M+H |
| *N*-Acetylhistidine | 4.83 | 138.8 | M+H |
| Phospho-L-arginine | 6.45 | 150.76 | M+H |
| Maltotriose hydrate | 6.09 | 211.48 | M+K |
| Maltotriose hydrate | 6.09 | 210.81 | M+Na |
| Allyl-glucosinolate | 3.21 | 168.74 | M+H |
| 5-Methylcytidine | 3.06 | 159.97 | M+Na |
| 5-Methylcytidine | 3.06 | 152.43 | M+H |
| 5-Methylcytidine | 3.06 | 162.31 | M+K |
| Nicotinamide n-oxide | 1.58 | 121.53 | M+H |
| D-glucose | 4.36 | 135.56 | M+Na |
| Cholic acid | 0.59 | 197.08 | M+Na |
| Cholic acid | 0.59 | 198.94 | M+K |
| Flavin-adenine-dinucleotide | 6.12 | 247.76 | M+H |
| Flavin-adenine-dinucleotide | 6.12 | 264.01 | M+K |
| 1,3,7-Trimethyluric acid | 0.61 | 140.22 | M+H |
| 1,7-Dimethyluric acid | 1.51 | 135.33 | M+H |
| 1-Methyladenosine | 4 | 159.4 | M+H |
| 1-Methyladenosine | 4 | 168.26 | M+Na |
| Retinol | 0.4 | 169.71 | M+H |
| 2-keto-l-gulonic acid | 5.67 | 137.07 | M+Na |
| 5-Hydroxyindole-3-acetic acid | 0.58 | 135.29 | M+H |
| 3-Methyl-2-oxindole | 0.43 | 125.95 | M+H |
| 2-Deoxyguanosine | 3.06 | 166.45 | M+Na |
| 2-Deoxyguanosine | 3.06 | 166.66 | M+K |
| D-Tagatose | 0.58 | 129.8 | M+H |
| 17-Beta-Estradiol | 0.42 | 163.16 | M+H |
| Pantothenic acid | 1.78 | 149.08 | M+Na |
| Pantothenic acid | 1.78 | 145.9 | M+H |
| 5-hydroxytryptophan | 4.31 | 148.96 | M+Na |
| *N*-Myristoylglycine | 0.87 | 167.92 | M+H |
| 3-Hydroxykynurenine | 4.19 | 151.06 | M+Na |
| 3-hydroxykynurenine | 4.19 | 147.93 | M+H |
| Cholecalciferol | 0.36 | 210.38 | M+H |
| N-Acetyl carnosine | 5.23 | 155.81 | M+H |
| N-acetyl carnosine | 5.23 | 161.51 | M+K |
| N-acetyl carnosine | 5.23 | 159.19 | M+Na |
| Riboflavin | 2.71 | 189.23 | M+Na |
| Riboflavin | 2.71 | 183.99 | M+H |
| Riboflavin | 2.71 | 191.52 | M+K |
| Adenosine 3,5-cyclic monophosphate | 4.36 | 170.91 | M+H |
| 4-Amino-2-methylpyridine | 4.68 | 115.12 | M+H |
| 3,7-Dimethyluric acid | 1.68 | 133.82 | M+H |
| Glycyl-L-leucine | 0.43 | 139.71 | M+H |
| Xanthurenic acid | 3.19 | 135.76 | M+H |
| Glycoursodeoxycholic acid | 1.69 | 204.08 | M+K |
| Glycoursodeoxycholic acid | 1.69 | 200.53 | M+Na |
| Glycodeoxycholic acid | 1.87 | 200.1 | M+H |
| Glycodeoxycholic acid | 1.87 | 203.24 | M+K |
| Glycodeoxycholic acid | 1.87 | 202.04 | M+Na |
| Melatonin | 0.53 | 155.06 | M+Na |
| Melatonin | 0.53 | 150.33 | M+H |
| Esculin | 2.04 | 172.91 | M+H |
| Esculin | 2.04 | 182.35 | M+Na |
| Ascorbic acid | 0.54 | 129.72 | M+H |
| Isocaffeine | 0.6 | 134.67 | M+H |
| Isocaffeine | 0.6 | 143.89 | M+Na |
| Glycocholic acid | 3.17 | 204 | M+H |
| Glycocholic acid | 3.17 | 203 | M+Na |
| Glycocholic acid | 3.17 | 207.1 | M+K |
| Riboflavin 5-monophosphate Sodium Salt | 5.71 | 196.73 | M+H |
| Riboflavin 5-monophosphate Sodium Salt | 5.71 | 204.73 | M+K |
| Riboflavin 5-monophosphate Sodium Salt | 5.71 | 203.5 | M+Na |
| Cytidine | 3.45 | 157.24 | M+K |
| Cytidine | 3.45 | 154.53 | M+Na |
| Cytidine | 3.45 | 147.11 | M+H |
| Folic acid | 4.95 | 195.41 | M+H |
| Folic acid | 4.95 | 203.97 | M+K |
| Folic acid | 4.95 | 201.14 | M+Na |
| Tauroursodeoxycholic acid | 1.98 | 212.96 | M+Na |
| Tauroursodeoxycholic acid | 1.98 | 214.21 | M+K |
| 1-Methylxanthine | 0.82 | 127.75 | M+H |
| 2-Deoxyadenosine-5-monophosphate | 5.71 | 163.45 | M+H |
| 2-Deoxyadenosine-5-monophosphate | 5.71 | 168.72 | M+Na |
| 2-Deoxycytidine | 2.57 | 155.17 | M+K |
| 2-Deoxycytidine | 2.57 | 151.35 | M+Na |
| Adenosine | 1.8 | 155.8 | M+H |
| 2-*O*-methyladenosine | 1 | 158.96 | M+H |
| 4-Guanidinobutyric acid | 4.36 | 125.47 | M+H |
| 6-Methylnicotinamide | 0.57 | 122.08 | M+H |
| Etiocholanolone | 0.41 | 169.72 | M+H |
| Etiocholanolone | 0.41 | 174.6 | M+Na |
| Hypoxanthine | 1.32 | 120.32 | M+H |
| 1,9-Dimethyluric acid | 1.51 | 135.33 | M+H |
| Cytidine-2,3-cyclic-phosphate | 5.03 | 162.36 | M+Na |
| Cytidine-2,3-cyclic-phosphate | 5.03 | 158.12 | M+H |
| Cytidine-2,3-cyclic-phosphate | 5.03 | 163.5 | M+K |
| 1-Methyl-l-histidine | 5.49 | 135.22 | M+Na |
| 1-Methyl-l-histidine | 5.49 | 129.55 | M+H |
| 11-Ketotestosterone | 0.42 | 169.64 | M+H |
| Dehydrocholic acid | 0.47 | 192.15 | M+Na |
| Dehydrocholic acid | 0.47 | 193.7 | M+K |
| 3,4,5-Trimethoxycinnamic acid | 0.49 | 150.35 | M+H |
| 8-Hydroxy-2-deoxyguanosine | 3.35 | 162.68 | M+Na |
| Theophylline | 0.57 | 131.37 | M+H |
| Theophylline | 0.57 | 139.35 | M+Na |
| 5,6-Dimethylbenzimidazole | 0.61 | 127.89 | M+H |
| 1,3,5-Trimethoxybenzene | 0.39 | 128.83 | M+H |
| L-Phenylalanine | 3.54 | 137.02 | M+Na |
| Phloretin | 0.56 | 158.28 | M+H |
| 21-Hydroxyprogesterone | 0.42 | 181.2 | M+H |
| 3-Phenylpropionic acid | 0.46 | 123.36 | M+H |
| Inosine | 3.02 | 165.21 | M+K |
| Inosine | 3.02 | 164.97 | M+Na |
| Inosine | 3.02 | 152.98 | M+H |
| 3-Methyl-L-histidine | 5.98 | 130.83 | M+H |
| 3-Nitrotyrosine | 3.64 | 151.59 | M+H |
| Glutathione | 5.92 | 164.56 | M+H |
| Glutathione | 5.92 | 163.47 | M+Na |
| Glutathione | 5.92 | 167.32 | M+K |
| Tryptophol | 0.43 | 130.09 | M+H |
| Guanosine-5-monophosphate | 6.12 | 174.13 | M+H |
| Taurolithocholic acid | 0.66 | 210.87 | M+K |
| taurolithocholic acid | 0.66 | 208.83 | M+Na |
| *N-*Acetyl-L-arginine dihydrate | 4.91 | 146.25 | M+H |
| *N*-Acetyl-L-arginine dihydrate | 4.91 | 155.91 | M+Na |
| *N*-Acetyl-5-hydroxytryptamine | 0.67 | 146.71 | M+H |
| Ophthalmic acid | 5.89 | 160.88 | M+Na |
| Ophthalmic acid | 5.89 | 161.58 | M+H |
| Ophthalmic acid | 5.89 | 164.56 | M+K |
| 1,3-Dimethyluracil | 0.46 | 120.23 | M+H |
| alpha-Lactose | 5.65 | 172.43 | M+Na |
| alpha-Lactose | 5.65 | 174.69 | M+K |
| Hydrocortisone | 0.52 | 184.62 | M+H |
| *O*-Phospho-DL-serine | 4.36 | 138.54 | M+Na |
| Indole-3-lactic acid | 0.86 | 140.12 | M+H |
| Taurocyamine | 4.36 | 129.35 | M+H |
| Taurocyamine | 4.36 | 139.31 | M+Na |
| L-Nicotine | 2.36 | 134.4 | M+H |
| L-Glutathione disulphide | 6.4 | 220.4 | M+Na |
| L-Glutathione disulphide | 6.4 | 224.71 | M+H |
| L-Glutathione disulphide | 6.4 | 214.94 | M+K |
| Androstenedione | 0.41 | 167.82 | M+H |
| N-alpha-Acetyl-L-lysine | 5.09 | 141.07 | M+H |
| Adrenosterone | 0.41 | 168.99 | M+H |
| L-Cystathionine | 6.23 | 142.98 | M+H |
| Paraxanthine | 0.59 | 130.25 | M+H |
| Estrone | 0.42 | 162.78 | M+H |
| [(3R)-3-Hydroxyhexanoyl]-L-carnitine | 0.58 | 166.77 | M+H |
| L-Alanyl-L-glutamine | 5.87 | 151.04 | M+K |
| L-Alanyl-L-glutamine | 5.87 | 144.39 | M+H |
| L-Alanyl-L-glutamine | 5.87 | 148.63 | M+Na |
| 7-Methylxanthine | 1.3 | 126.06 | M+H |
| L-Homocitrulline | 5.47 | 140.7 | M+Na |
| Indole-3-pyruvic acid | 3.06 | 136.81 | M+H |
| *N*-Acetyl-d-mannosamine | 3.73 | 145.5 | M+Na |
| Quinoline-4-carboxylic acid | 2.06 | 129.63 | M+H |
| Cytosine | 3.44 | 112.85 | M+H |
| 1-Methylguanine | 1.7 | 127.45 | M+H |
| 1-Methylguanine | 1.7 | 135 | M+Na |

**Table S2:** Measured authentic standards (-ve ESI) used to generate an in-house database for searching via Progenesis QI.

| **Name** | **Retention Time (min)** | **CCS (angstrom^2)** | **Adduct** |
| --- | --- | --- | --- |
| Uridine-5-monophosphate | 5.93 | 157.33 | M-H |
| 1-Methylguanine | 1.71 | 119.26 | M-H |
| 3-(2-Hydroxyphenyl)propanoic acid | 0.54 | 125.42 | M-H |
| N-(2-Furoyl)glycine | 2.18 | 126.14 | M-H |
| Estriol | 0.43 | 175.87 | M+CH3COO |
| Pimelic acid | 0.9 | 123.11 | M-H |
| *N-A*cetyl-5-hydroxytryptamine | 0.56 | 145.27 | M-H |
| Xanthosine-dihydrate | 4.07 | 152.98 | M-H |
| gamma-Glutamylalanine | 5.91 | 136.86 | M-H |
| L-Phenylalanine | 3.55 | 131.09 | M-H |
| N-*M*yristoylglycine | 0.54 | 178.12 | M-H |
| Ophthalmic acid | 5.89 | 156.47 | M-H |
| L-Tryptophan | 2.96 | 160.48 | M+CH3COO |
| *N*-4-acetylcytidine | 1.94 | 159.05 | M-H |
| Pantothenic acid | 1.89 | 142.18 | M-H |
| 3-Methyladipic acid | 1.03 | 123.39 | M-H |
| Glycyl-L-valine | 4.72 | 132.29 | M-H |
| Phenylacetylglutamine | 2.99 | 160.16 | M-H |
| 5-Methyluridine | 1.63 | 150.92 | M-H |
| 5-Methyluridine | 1.63 | 156.24 | M+Cl |
| 2-Deoxyadenosine-5-monophosphate | 5.82 | 166.71 | M-H |
| Dehydroepiandrosterone-sulfate | 0.54 | 192.72 | M-H |
| Cytidine | 3.42 | 143.55 | M-H |
| Cytidine | 3.42 | 153.16 | M+Cl |
| Cellobiose | 5.26 | 164.8 | M-H |
| 4-Methylhippuric acid | 1.44 | 140.49 | M-H |
| Inosine-5-monophosphate (sodium salt hydrate) | 6.01 | 167.82 | M-H |
| Cytidine-5-monophosphate | 6.15 | 159.24 | M-H |
| Pyridoxine | 1.38 | 126.12 | M-H |
| DL-tryptophan | 3.55 | 143.26 | M-H |
| Cytidine-2,3-cyclic-phosphate | 5.02 | 158.57 | M-H |
| L-alanyl-l-glutamine | 5.86 | 143.78 | M-H |
| Adenosine-5-triphosphate | 6.68 | 186.94 | M-H |
| Uridine | 1.93 | 143.72 | M-H |
| Carnosine | 6.08 | 146.63 | M-H |
| N-Acetyl carnosine | 5.23 | 156.98 | M-H |
| 3-Chloro-L-tyrosine | 3.94 | 142.15 | M-H |
| Melatonin | 0.44 | 150 | M-H |
| Xanthine | 4.04 | 111.3 | M-H |
| Cholic acid | 0.64 | 201.67 | M-H |
| Cholic acid | 0.64 | 201.98 | M+Cl |
| D-Saccharic Acid | 6.21 | 126.44 | M-H |
| N-Acetyl-L-methionine | 1.78 | 136.42 | M-H |
| N-Acetylhistidine | 4.83 | 134.23 | M-H |
| Flavin-adenine-dinucleotide | 6.11 | 238.94 | M-H |
| Taurocyamine | 4.34 | 120.68 | M-H |
| 4-Hydroxy-3-methoxymandelic acid | 2.22 | 135.61 | M-H |
| 3-Hydroxycinnamic acid | 0.58 | 122.28 | M-H |
| Hydrocortisone | 0.57 | 187.33 | M+Cl |
| Inosine | 2.88 | 151.76 | M-H |
| Cysteic acid | 5.78 | 114.88 | M-H |
| DL-3,4-Dihydroxymandelic acid | 4.04 | 129.01 | M-H |
| Tricarballylic acid | 4.37 | 118.78 | M-H |
| gamma-Glutamylcysteine | 5.9 | 144.47 | M-H |
| Riboflavin 5-Monophosphate Sodium Salt | 5.78 | 194.41 | M-H |
| *N*-methyl-L-phenylalanine | 2.83 | 135.36 | M-H |
| Saccharin sodium dihydrate | 0.59 | 122.28 | M-H |
| Ursodeoxycholic acid | 0.57 | 206.71 | M-H |
| Taurodeoxycholic acid | 2.09 | 205.28 | M-H |
| Decanoylcarnitine | 0.44 | 184.1 | M+Cl |
| Adenosine-monophosphate | 5.92 | 168.62 | M-H |
| Beta-Glycerophophate | 5.68 | 119.21 | M-H |
| Biotin | 1.24 | 149.85 | M-H |
| Glycyl-L-tyrosine | 5 | 150.17 | M-H |
| Sarcosyl-glycyl-glycine | 5.76 | 138.85 | M-H |
| Maltotriose hydrate | 6.06 | 205.6 | M-H |
| Maltotriose hydrate | 6.06 | 208.47 | M+Cl |
| Indole-3-butyric acid | 0.47 | 145.74 | M-H |
| Gallic acid | 2.19 | 116.4 | M-H |
| Hexadecanedioic acid | 0.47 | 168.36 | M-H |
| L-3-Phenyllactic acid | 0.74 | 127.96 | M-H |
| Indole-3-lactic acid | 1.17 | 142.18 | M-H |
| 5-Uridylic acid-disodium-salt | 5.94 | 157.17 | M-H |
| Cholesterol sulfate | 0.46 | 237.99 | M-H |
| D-glucose-6-phosphate | 6.22 | 141.83 | M-H |
| 3-Methyl-L-histidine | 5.99 | 129.31 | M-H |
| Galactaric acid | 6.25 | 126.65 | M-H |
| Suberic acid | 0.67 | 128.42 | M-H |
| 7-Methylxanthine | 1.19 | 117.7 | M-H |
| 1-Methylxanthine | 1 | 117.37 | M-H |
| 2,2-Dimethylsuccinic acid | 0.88 | 118.19 | M-H |
| 7-Methylguanine | 2.05 | 122.37 | M-H |
| Indole-3-carboxylic acid | 0.53 | 117.38 | M-H |
| 4-Hydroxyphenylglycolic acid | 2.63 | 123.94 | M-H |
| Isocaffeine | 1.07 | 134.87 | M-H |
| Phenylacetylglycine | 1.42 | 141.05 | M-H |
| L-Homocitrulline | 5.48 | 133.41 | M-H |
| 2,3-Dihydroxybenzoic acid | 1.01 | 112.15 | M-H |
| Guanosine-5-monophosphate | 6.14 | 163.98 | M-H |
| Allyl-glucosinolate | 3.18 | 168.36 | M-H |
| 2-Deoxyguanosine-5-monophosphate | 6.05 | 163.52 | M-H |
| Alpha-lactose | 5.43 | 171.45 | M+Cl |
| D-Mannitol | 4.35 | 125.61 | M-H |
| Glycodeoxycholic acid | 1.82 | 198.46 | M-H |
| 2-Methylhippuric acid | 1.31 | 139.9 | M-H |
| L-Thyroxine | 2.24 | 208.49 | M-H |
| Sebacic acid | 0.56 | 137.93 | M-H |
| Sebacic acid | 0.56 | 156.74 | M+Cl |
| Glycoursodeoxycholic acid | 1.89 | 199.51 | M-H |
| Tetradecanedioic acid | 0.48 | 158.95 | M-H |
| Esculin | 2 | 169.01 | M-H |
| DL-Arginine | 6.03 | 129.26 | M-H |
| 3-Methylhippuric acid | 1.41 | 141.71 | M-H |
| 2-Deoxyguanosine | 3.07 | 153.31 | M-H |
| Gentisic acid | 1.34 | 114.31 | M-H |
| Deoxycholic acid | 0.56 | 200.44 | M-H |
| L-Glutathione disulphide | 6.39 | 218.04 | M-H |
| *N*-Acetylanthranilic acid | 0.6 | 126.19 | M-H |
| 3,7-Dimethyluric acid | 1.73 | 128.2 | M-H |
| Uric acid | 4.35 | 115.5 | M-H |
| *N*-Acetyl-L-tyrosine | 2.54 | 145.91 | M-H |
| Adenosine 3,5-cyclic monophosphate | 4.36 | 166.96 | M-H |
| Tauroursodeoxycholic acid | 2.03 | 206.44 | M-H |
| L-cystine | 6.25 | 137.84 | M-H |
| 2,6-Dihydroxybenzoic acid | 1.11 | 112.2 | M-H |
| *N*-Acetyl-L-arginine dihydrate | 4.91 | 142.54 | M-H |
| Piperidine-2,6-dicarboxylic acid | 5.96 | 124.21 | M-H |
| 4-Pyridoxic acid | 0.74 | 124.09 | M-H |
| Chenodeoxycholic acid | 0.56 | 198.74 | M+Cl |
| Chenodeoxycholic acid | 0.56 | 207.3 | M-H |
| 1,3,7-Trimethyluric acid | 0.7 | 134.23 | M-H |
| Glycochenodeoxycholic acid | 1.89 | 199.51 | M-H |
| 3-Indolepropionic acid | 0.68 | 139.46 | M-H |
| 1,9-Dimethyluric acid | 1.53 | 127.91 | M-H |
| Phenylpropionylglycine | 1.08 | 148.69 | M-H |
| *N*-Isovaleroylglycine | 1.64 | 132.54 | M-H |
| 3-Nitrotyrosine | 3.65 | 142.09 | M-H |
| 3(3-Hydroxyphenyl)propionic acid | 0.54 | 125.42 | M-H |
| Taurocholic acid | 2.94 | 206.02 | M-H |
| DL-*p*-Hydroxyphenyllactic acid | 2.08 | 132.64 | M-H |
| Podocarpic acid | 0.45 | 162.91 | M-H |
| Naringenin | 0.46 | 157.04 | M-H |
| 5-alpha-Cholestan-3-one | 0.41 | 207.77 | M+Cl |
| *N*-Acetyl-neuraminic acid | 5.79 | 158.2 | M-H |
| L-Hmocystine | 6.1 | 147.82 | M-H |
| *N*-Acetyl-L-glutamine | 4.36 | 131.88 | M-H |
| 1-Ketopicnic Acid | 0.51 | 135.05 | M-H |
| *N*-Acetyl-L-proline | 3.43 | 124.93 | M-H |
| Theophylline | 0.62 | 124.93 | M-H |
| Cysteinyl-glycine | 5.03 | 129.5 | M-H |
| Nicotinuric acid | 3.75 | 130.23 | M-H |
| 5-Hydroxytryptophan | 4.3 | 147.62 | M-H |
| Tridecanedioic acid | 0.5 | 154.23 | M-H |
| Glycyl-L-leucine | 4.33 | 139.04 | M-H |
| *N*-Acetyl-DL-tryptophan | 1.41 | 153.22 | M-H |
| Noradrenaline-bitartrate | 3.74 | 126.73 | M-H |
| Guanine | 3.8 | 114.77 | M-H |
| *N*-Acetyl-L-aspartic acid | 4.34 | 123.15 | M-H |
| Ferulic acid | 0.52 | 131.56 | M-H |
| Glycocholic acid | 3.2 | 200.46 | M-H |
| Guanosine | 3.81 | 155.96 | M-H |
| Guanosine | 3.81 | 160.96 | M+Cl |
| Hyocholic acid | 0.57 | 208.41 | M-H |
| Hyocholic acid | 0.57 | 211.02 | M+CH3COO |
| 3,4,5-Trimethoxycinnamic acid | 0.48 | 157.55 | M-H |
| riboflavin | 2.71 | 183.12 | M-H |
| alpha-D-Galactose 1-phosphate | 6.17 | 143.32 | M-H |
| D-Isoascorbic Acid | 4.3 | 119.58 | M-H |
| DL-Phenylalanine | 3.55 | 130.33 | M-H |
| 10-Hydroxydecanoic-acid | 0.48 | 139.84 | M-H |
| Homovanillic acid | 1.63 | 128.5 | M-H |
| 8-hydroxy-2-deoxyguanosine | 3.31 | 154.35 | M-H |
| *N*-alpha-Acetyl-L-lysine | 5.09 | 140.19 | M-H |
| 2-Aminobenzenesulfonic acid | 1.21 | 121.54 | M-H |
| 11-Hydroxyundecanoic acid | 0.46 | 145.07 | M-H |
| Sucrose | 5.09 | 169.15 | M+Cl |
| Sucrose | 5.09 | 164.56 | M-H |
| 3-Methylxanthine | 1.19 | 117.7 | M-H |
| Dodecanedioic acid | 0.51 | 149.03 | M-H |
| 5-Methyl-2-deoxycytidine | 2.35 | 148.61 | M-H |
| *N*-acetyl-L-leucine | 1.13 | 134.46 | M-H |
| 4-Hydroxycinnamic acid | 0.57 | 122.03 | M-H |
| Isoxanthopterin | 3.02 | 120.47 | M-H |
| 1,7-Dimethyluric acid | 1.53 | 127.91 | M-H |
| Dehydrocholic acid | 0.46 | 204.44 | M-H |
| *N-*Formyl-L-methionine | 2.18 | 129.54 | M-H |
| *N*-Acetyl-L-glutamic acid | 4.33 | 129.25 | M-H |
| Phloretin | 0.56 | 158.47 | M-H |
| Uridine-5-monophosphate | 5.93 | 157.33 | M-H |
| 1-Methylguanine | 1.71 | 119.26 | M-H |
| 3-(2-Hydroxyphenyl)propanoic acid | 0.54 | 125.42 | M-H |
| *N*-(2-Furoyl)glycine | 2.18 | 126.14 | M-H |
| Estriol | 0.43 | 175.87 | M+CH3COO |
| Pimelic acid | 0.9 | 123.11 | M-H |
| *N*-Acetyl-5-hydroxytryptamine | 0.56 | 145.27 | M-H |
| Xanthosine-dihydrate | 4.07 | 152.98 | M-H |
| gamma-Glutamylalanine | 5.91 | 136.86 | M-H |
| L-Phenylalanine | 3.55 | 131.09 | M-H |
| *N*-Myristoylglycine | 0.54 | 178.12 | M-H |
| Ophthalmic acid | 5.89 | 156.47 | M-H |
| L-tryptophan | 2.96 | 160.48 | M+CH3COO |
| *N*-4-Acetylcytidine | 1.94 | 159.05 | M-H |
| Pantothenic acid | 1.89 | 142.18 | M-H |
| 3-Methyladipic acid | 1.03 | 123.39 | M-H |
| Glycyl-L-valine | 4.72 | 132.29 | M-H |
| Phenylacetylglutamine | 2.99 | 160.16 | M-H |
| 5-Methyluridine | 1.63 | 150.92 | M-H |
| 5-Methyluridine | 1.63 | 156.24 | M+Cl |
| 2-Deoxyadenosine-5-monophosphate | 5.82 | 166.71 | M-H |
| Dehydroepiandrosterone-sulfate | 0.54 | 192.72 | M-H |
| Cytidine | 3.42 | 143.55 | M-H |
| Cytidine | 3.42 | 153.16 | M+Cl |
| Cellobiose | 5.26 | 164.8 | M-H |
| 4-Methylhippuric acid | 1.44 | 140.49 | M-H |
| Inosine-5-monophosphate (sodium salt hydrate) | 6.01 | 167.82 | M-H |
| Cytidine-5-monophosphate | 6.15 | 159.24 | M-H |
| Ppyridoxine | 1.38 | 126.12 | M-H |
| DL-tryptophan | 3.55 | 143.26 | M-H |
| Cytidine-2,3-cyclic-phosphate | 5.02 | 158.57 | M-H |
| L-alanyl-L-glutamine | 5.86 | 143.78 | M-H |
| Adenosine-5-triphosphate | 6.68 | 186.94 | M-H |
| Uridine | 1.93 | 143.72 | M-H |
| Carnosine | 6.08 | 146.63 | M-H |
| *N*-acetyl carnosine | 5.23 | 156.98 | M-H |
| 3-Chloro-L-tyrosine | 3.94 | 142.15 | M-H |
| melatonin | 0.44 | 150 | M-H |
| Xanthine | 4.04 | 111.3 | M-H |
| Cholic acid | 0.64 | 201.67 | M-H |
| Cholic acid | 0.64 | 201.98 | M+Cl |
| D-Saccharic Acid | 6.21 | 126.44 | M-H |
| *N*-Acetyl-L-methionine | 1.78 | 136.42 | M-H |
| N-Acetylhistidine | 4.83 | 134.23 | M-H |
| Flavin-adenine-dinucleotide | 6.11 | 238.94 | M-H |
| Taurocyamine | 4.34 | 120.68 | M-H |
| 4-Hydroxy-3-Methoxymandelic Acid | 2.22 | 135.61 | M-H |
| 3-Hydroxycinnamic acid | 0.58 | 122.28 | M-H |
| Hydrocortisone | 0.57 | 187.33 | M+Cl |
| Inosine | 2.88 | 151.76 | M-H |
| Cysteic acid | 5.78 | 114.88 | M-H |
| DL-3,4-Dihydroxymandelic acid | 4.04 | 129.01 | M-H |
| Tricarballylic acid | 4.37 | 118.78 | M-H |
| gamma-Glutamylcysteine | 5.9 | 144.47 | M-H |
| Riboflavin 5-Monophosphate Sodium Salt | 5.78 | 194.41 | M-H |
| *N*-methyl-L-phenylalanine | 2.83 | 135.36 | M-H |
| Saccharin sodium dihydrate | 0.59 | 122.28 | M-H |
| Ursodeoxycholic acid | 0.57 | 206.71 | M-H |
| Taurodeoxycholic acid | 2.09 | 205.28 | M-H |
| Decanoylcarnitine | 0.44 | 184.1 | M+Cl |
| Adenosine-monophosphate | 5.92 | 168.62 | M-H |
| beta-Glycerophophate | 5.68 | 119.21 | M-H |
| Biotin | 1.24 | 149.85 | M-H |
| Glycyl-l-tyrosine | 5 | 150.17 | M-H |
| Sarcosyl-glycyl-glycine | 5.76 | 138.85 | M-H |
| Maltotriose hydrate | 6.06 | 205.6 | M-H |
| Maltotriose hydrate | 6.06 | 208.47 | M+Cl |
| Indole-3-butyric acid | 0.47 | 145.74 | M-H |
| Gallic acid | 2.19 | 116.4 | M-H |
| Hexadecanedioic acid | 0.47 | 168.36 | M-H |
| L-3-Phenyllactic acid | 0.74 | 127.96 | M-H |
| Indole-3-lactic acid | 1.17 | 142.18 | M-H |
| 5-Uridylic acid-disodium-salt | 5.94 | 157.17 | M-H |
| Cholesterol sulfate | 0.46 | 237.99 | M-H |
| D-glucose-6-phosphate | 6.22 | 141.83 | M-H |
| 3-Methyl-L-histidine | 5.99 | 129.31 | M-H |
| Galactaric acid | 6.25 | 126.65 | M-H |
| Suberic acid | 0.67 | 128.42 | M-H |
| 7-Methylxanthine | 1.19 | 117.7 | M-H |
| 1-Methylxanthine | 1 | 117.37 | M-H |
| 2,2-Dimethylsuccinic acid | 0.88 | 118.19 | M-H |
| 7-Methylguanine | 2.05 | 122.37 | M-H |
| Indole-3-carboxylic acid | 0.53 | 117.38 | M-H |
| 4-Hydroxyphenylglycolic acid | 2.63 | 123.94 | M-H |
| Isocaffeine | 1.07 | 134.87 | M-H |
| Phenylacetylglycine | 1.42 | 141.05 | M-H |
| L-Homocitrulline | 5.48 | 133.41 | M-H |
| 2,3-Dihydroxybenzoic acid | 1.01 | 112.15 | M-H |
| Guanosine-5-monophosphate | 6.14 | 163.98 | M-H |
| Allyl-glucosinolate | 3.18 | 168.36 | M-H |
| 2-Deoxyguanosine-5-monophosphate | 6.05 | 163.52 | M-H |
| alpha-Lactose | 5.43 | 171.45 | M+Cl |
| D-mannitol | 4.35 | 125.61 | M-H |
| Glycodeoxycholic acid | 1.82 | 198.46 | M-H |
| 2-Methylhippuric acid | 1.31 | 139.9 | M-H |
| L-Thyroxine | 2.24 | 208.49 | M-H |
| Sebacic acid | 0.56 | 137.93 | M-H |
| Sebacic acid | 0.56 | 156.74 | M+Cl |
| Glycoursodeoxycholic acid | 1.89 | 199.51 | M-H |
| tetradecanedioic acid | 0.48 | 158.95 | M-H |
| Esculin | 2 | 169.01 | M-H |
| DL-Arginine | 6.03 | 129.26 | M-H |
| 3-Methylhippuric acid | 1.41 | 141.71 | M-H |
| 2-Deoxyguanosine | 3.07 | 153.31 | M-H |
| Gentisic acid | 1.34 | 114.31 | M-H |
| Deoxycholic acid | 0.56 | 200.44 | M-H |
| L-Glutathione disulphide | 6.39 | 218.04 | M-H |
| *N*-Acetylanthranilic acid | 0.6 | 126.19 | M-H |
| 3,7-Dimethyluric acid | 1.73 | 128.2 | M-H |
| Uric acid | 4.35 | 115.5 | M-H |
| *N*-acetyl-L-tyrosine | 2.54 | 145.91 | M-H |
| Adenosine 3,5-cyclic monophosphate | 4.36 | 166.96 | M-H |
| Tauroursodeoxycholic acid | 2.03 | 206.44 | M-H |
| L-cystine | 6.25 | 137.84 | M-H |
| 2,6-Dihydroxybenzoic acid | 1.11 | 112.2 | M-H |
| *N*-Acetyl-L-arginine dihydrate | 4.91 | 142.54 | M-H |
| Piperidine-2,6-dicarboxylic acid | 5.96 | 124.21 | M-H |
| 4-Pyridoxic acid | 0.74 | 124.09 | M-H |
| Chenodeoxycholic acid | 0.56 | 198.74 | M+Cl |
| Chenodeoxycholic acid | 0.56 | 207.3 | M-H |
| 1,3,7-Trimethyluric acid | 0.7 | 134.23 | M-H |
| glycochenodeoxycholic acid | 1.89 | 199.51 | M-H |
| 3-Indolepropionic acid | 0.68 | 139.46 | M-H |
| 1,9-Dimethyluric acid | 1.53 | 127.91 | M-H |
| Phenylpropionylglycine | 1.08 | 148.69 | M-H |
| *N*-isovaleroylglycine | 1.64 | 132.54 | M-H |
| 3-Nitrotyrosine | 3.65 | 142.09 | M-H |
| 3(3-Hydroxyphenyl)propionic acid | 0.54 | 125.42 | M-H |
| Taurocholic acid | 2.94 | 206.02 | M-H |
| DL-*p*-Hydroxyphenyllactic acid | 2.08 | 132.64 | M-H |
| Podocarpic acid | 0.45 | 162.91 | M-H |
| Naringenin | 0.46 | 157.04 | M-H |
| 5-alpha-Cholestan-3-one | 0.41 | 207.77 | M+Cl |
| N-Acetyl-neuraminic acid | 5.79 | 158.2 | M-H |
| L-Homocystine | 6.1 | 147.82 | M-H |
| *N*-Acetyl-L-Glutamine | 4.36 | 131.88 | M-H |
| 1-Ketopicnic Acid | 0.51 | 135.05 | M-H |
| *N*-Acetyl-L-proline | 3.43 | 124.93 | M-H |
| Theophylline | 0.62 | 124.93 | M-H |
| Cysteinyl-glycine | 5.03 | 129.5 | M-H |
| Nicotinuric acid | 3.75 | 130.23 | M-H |
| 5-hydroxytryptophan | 4.3 | 147.62 | M-H |
| Tridecanedioic acid | 0.5 | 154.23 | M-H |
| Glycyl-L-leucine | 4.33 | 139.04 | M-H |
| *N*-Acetyl-DL-tryptophan | 1.41 | 153.22 | M-H |
| Noradrenaline-bitartrate | 3.74 | 126.73 | M-H |
| Guanine | 3.8 | 114.77 | M-H |
| *N*-Acetyl-L-aspartic acid | 4.34 | 123.15 | M-H |
| Ferulic acid | 0.52 | 131.56 | M-H |
| Glycocholic acid | 3.2 | 200.46 | M-H |
| Guanosine | 3.81 | 155.96 | M-H |
| Guanosine | 3.81 | 160.96 | M+Cl |
| Hyocholic acid | 0.57 | 208.41 | M-H |
| Hyocholic acid | 0.57 | 211.02 | M+CH3COO |
| 3,4,5-Trimethoxycinnamic acid | 0.48 | 157.55 | M-H |
| Riboflavin | 2.71 | 183.12 | M-H |
| alpha-d-galactose 1-phosphate | 6.17 | 143.32 | M-H |
| D-Isoascorbic Acid | 4.3 | 119.58 | M-H |
| dl-phenylalanine | 3.55 | 130.33 | M-H |
| 10-hydroxydecanoic-acid | 0.48 | 139.84 | M-H |
| homovanillic acid | 1.63 | 128.5 | M-H |
| 8-hydroxy-2-deoxyguanosine | 3.31 | 154.35 | M-H |
| n-alpha-acetyl-l-lysine | 5.09 | 140.19 | M-H |
| 2-aminobenzenesulfonic acid | 1.21 | 121.54 | M-H |
| 11-hydroxyundecanoic acid | 0.46 | 145.07 | M-H |
| sucrose | 5.09 | 169.15 | M+Cl |
| sucrose | 5.09 | 164.56 | M-H |
| 3-methylxanthine | 1.19 | 117.7 | M-H |
| dodecanedioic acid | 0.51 | 149.03 | M-H |
| 5-Methyl-2-deoxycytidine | 2.35 | 148.61 | M-H |
| n-acetyl-l-leucine | 1.13 | 134.46 | M-H |
| 4-hydroxycinnamic acid | 0.57 | 122.03 | M-H |
| isoxanthopterin | 3.02 | 120.47 | M-H |
| 1,7-dimethyluric acid | 1.53 | 127.91 | M-H |
| dehydrocholic acid | 0.46 | 204.44 | M-H |
| N-Formyl-L-methionine | 2.18 | 129.54 | M-H |
| n-acetyl-l-glutamic acid | 4.33 | 129.25 | M-H |
| phloretin | 0.56 | 158.47 | M-H |


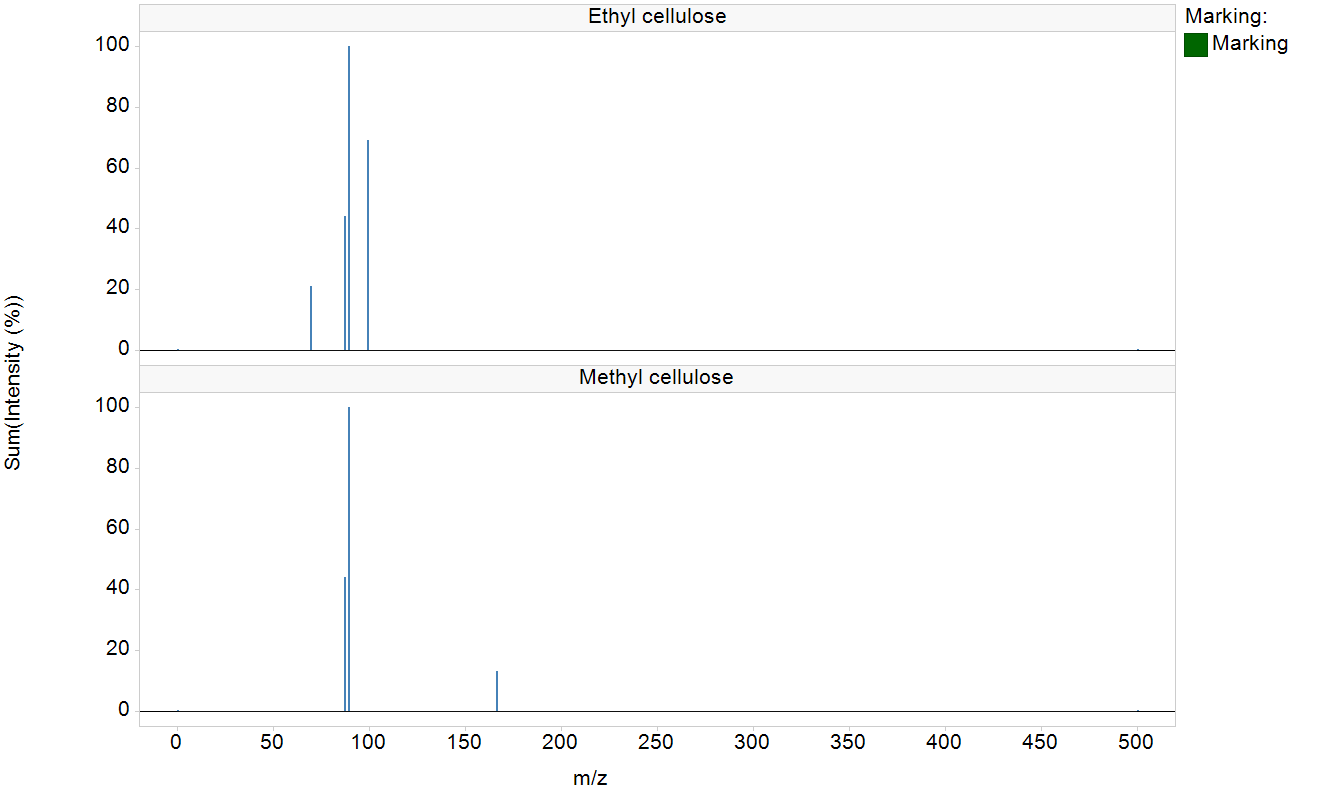


**Figure S1:** Characteristic fragmentation spectra obtained from the contaminating polymer peaks shown in **Figure 1** due to methyl and ethyl cellulose (precursor m/z 455.2485; mass error = 0.44 ppm; t_R_ = 1.0 min (ESI+)). All of the contaminating peaks showed similar fragments.

**Vehicle Oral**


**Figure S2:** Unsupervised principal component analysis (PCA) and associated heatmap (+ ve ESI) relating to the vehicle (**A**) and oral (**B**) gefitinib-dosed groups. The PCA analysis includes all features adhering to a CV filtering of <30%, whilst the heatmaps (A, B) correspond to the top 100 features based on ANOVA t-test. Heatmaps were constructed using Euclidean distance and Ward clustering.

**Figure S3:** Unsupervised principal component analysis (PCA) and associated heatmap (-ve ESI) relating to the vehicle and oral dosed groups. The PCA analysis includes all features adhering to a CV filtering of <30%, whilst the heatmaps correspond to the top 100 features based on ANOVA t-test. Heatmaps were constructed using Euclidean distance and Ward clustering.


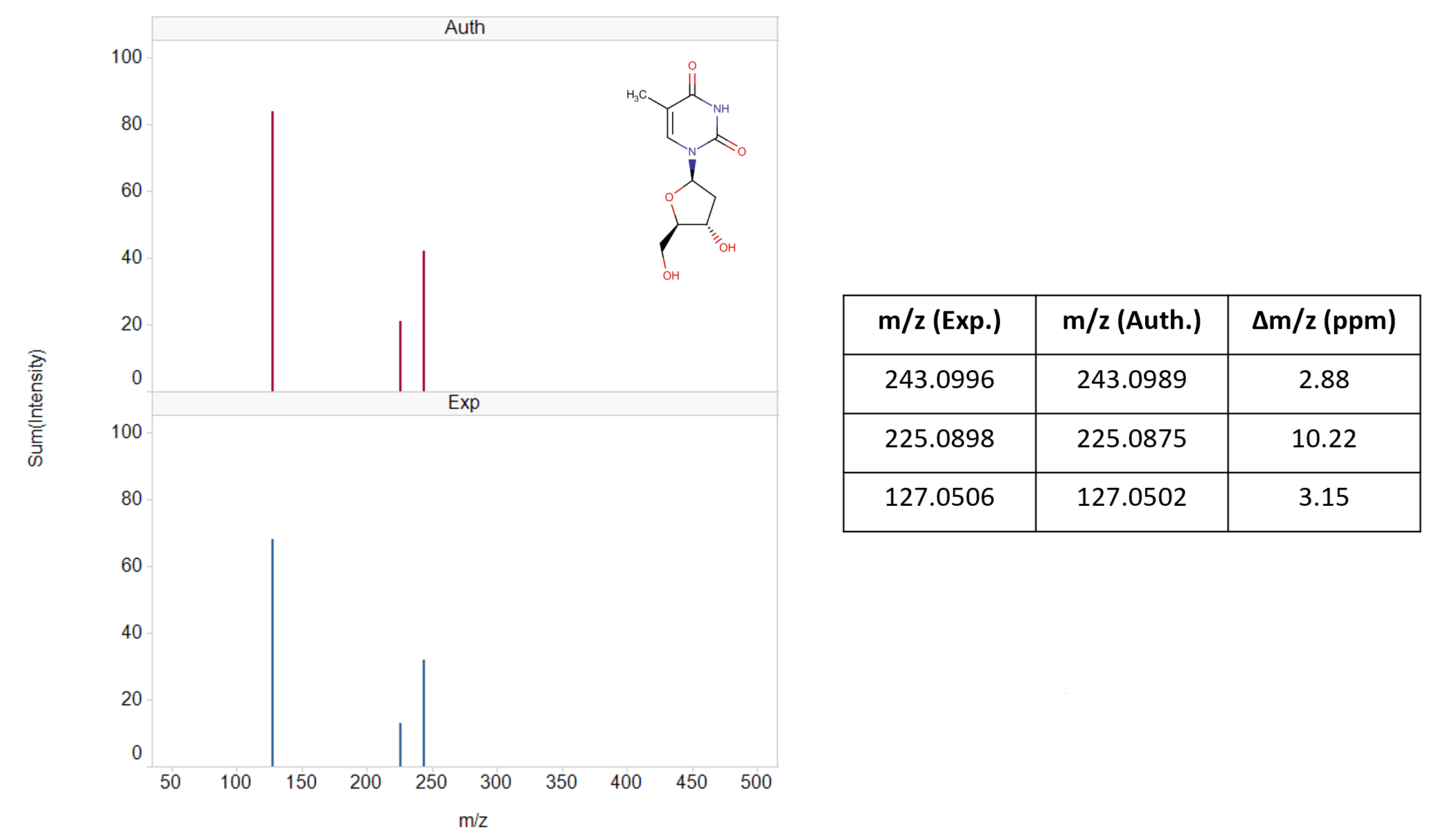


**Figure S4:** MS/MS spectra for thymidine comparing experimentally (Exp) derived data against its authentic standard (Auth). Spectra corresponding with the authentic standard (upper spectrum; red peaks) and Progenesis QI identified urine metabolite (lower spectrum; blue peaks) are shown. The difference in mass observed between the precursor and fragment ions of the experimental and authentic standard are represented as ppm in the associated table.

**Figure S5:** MS/MS spectra for myristoylglycine comparing experimentally (Exp) derived data against its authentic standard (Auth). Spectra corresponding with the authentic standard (upper spectrum; red peaks) and Progenesis QI identified urine metabolite (lower spectrum; blue peaks) are shown. The difference in mass observed between the precursor and fragment ions of the experimental and authentic standard are represented as ppm in the associated table.

**Figure S6:** MS/MS spectra for acetylcarnitine comparing experimentally (Exp) derived data against its authentic standard (Auth). Spectra corresponding with the authentic standard (upper spectrum; red peaks) and Progenesis QI identified urine metabolite (lower spectrum; blue peaks) are shown. The difference in mass observed between the precursor and fragment ions of the experimental and authentic standard are represented as ppm in the associated table.


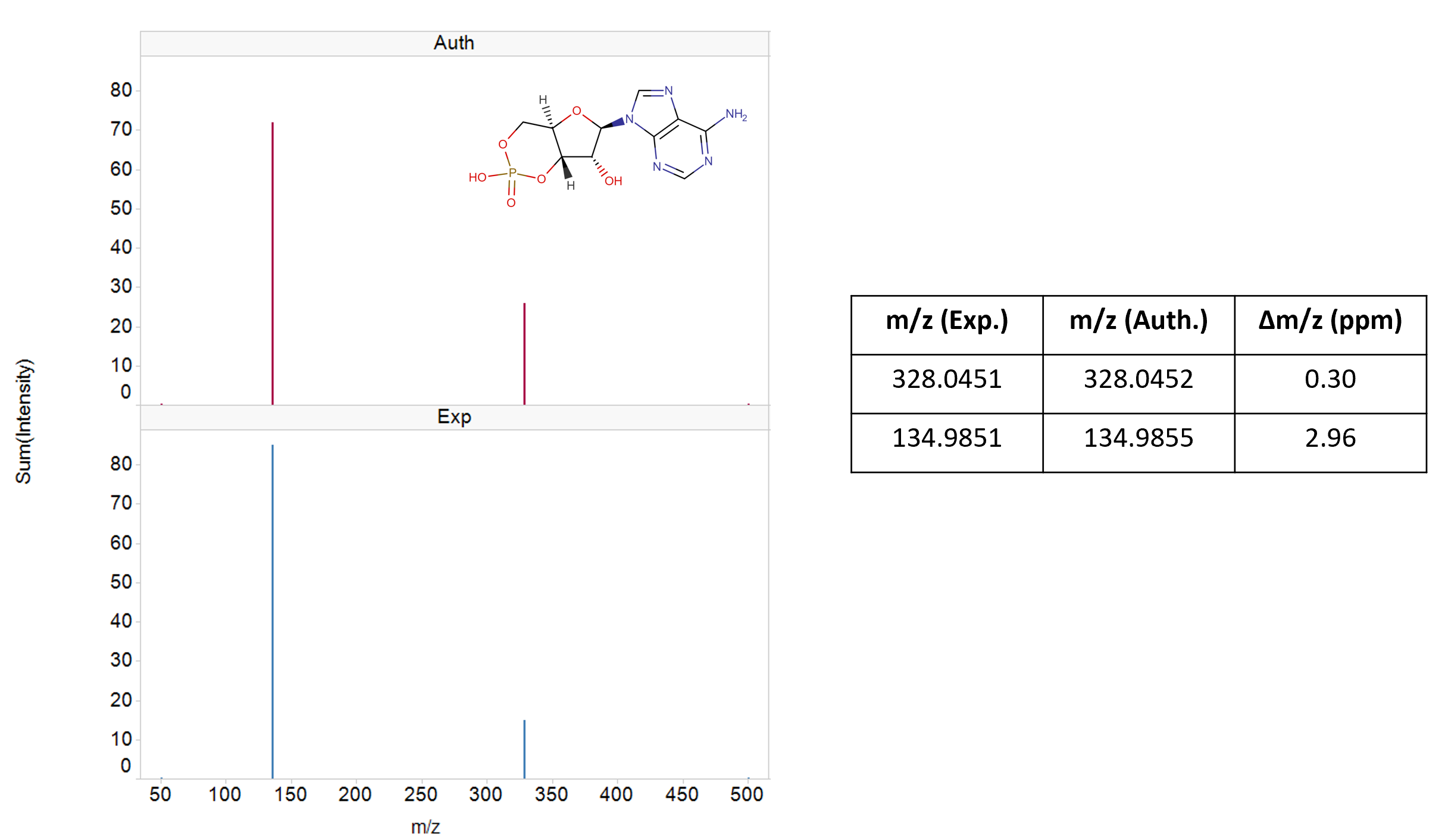


**Figure S7:** MS/MS spectra for cAMP comparing experimentally (Exp) derived data against its authentic standard (Auth). Spectra corresponding with the authentic standard (upper spectrum; red peaks) and Progenesis QI identified urine metabolite (lower spectrum; blue peaks) are shown. The difference in mass observed between the precursor and fragment ions of the experimental and authentic standard are represented as ppm in the associated table.


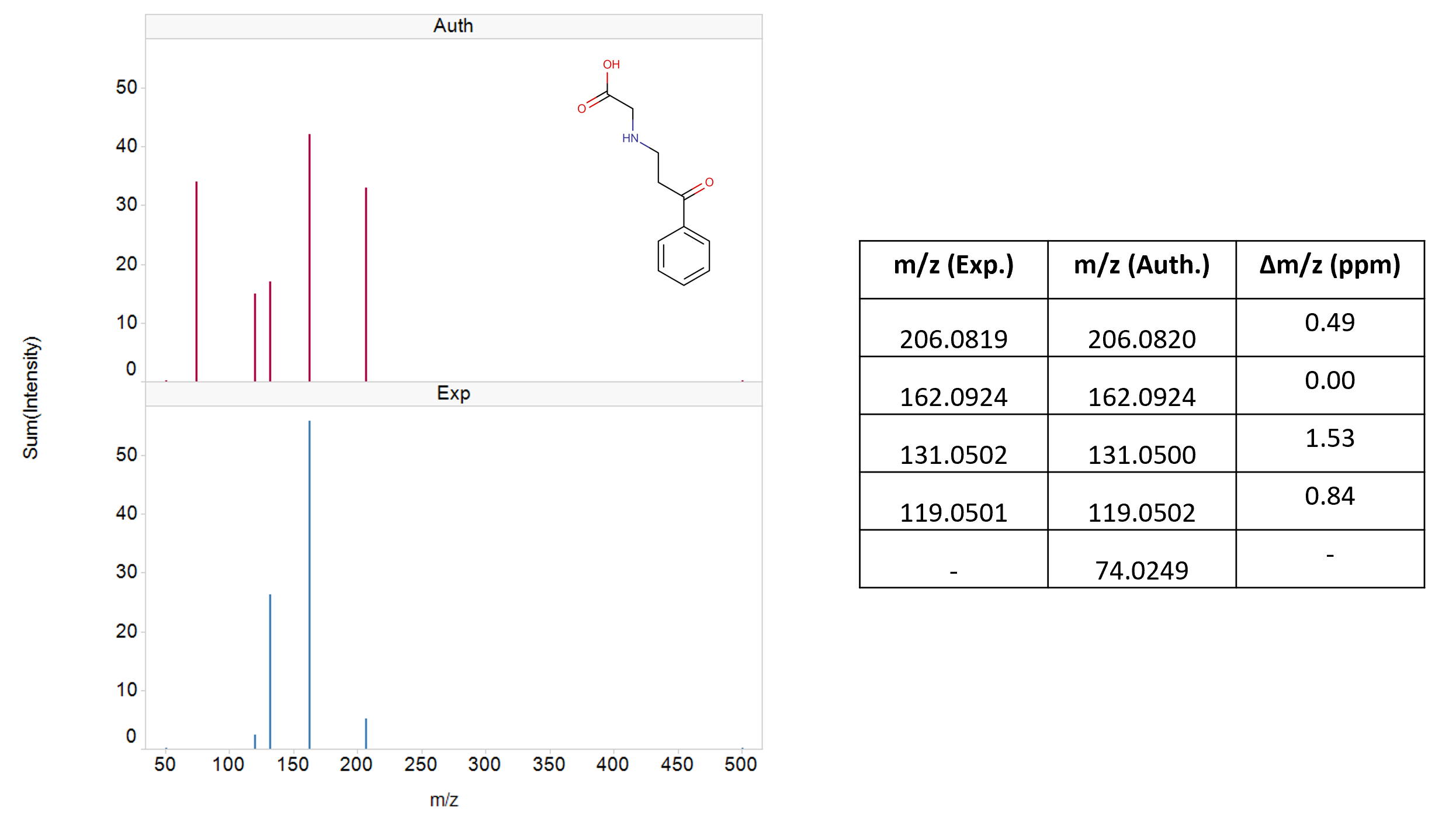


**Figure S8:** MS/MS spectra for 3-phenylpropionylglycine comparing experimentally (Exp) derived data against its authentic standard (Auth). Spectra corresponding with the authentic standard (upper spectrum; red peaks) and Progenesis QI identified urine metabolite (lower spectrum; blue peaks) are shown. The difference in mass observed between the precursor and fragment ions of the experimental and authentic standard are represented as ppm in the associated table.


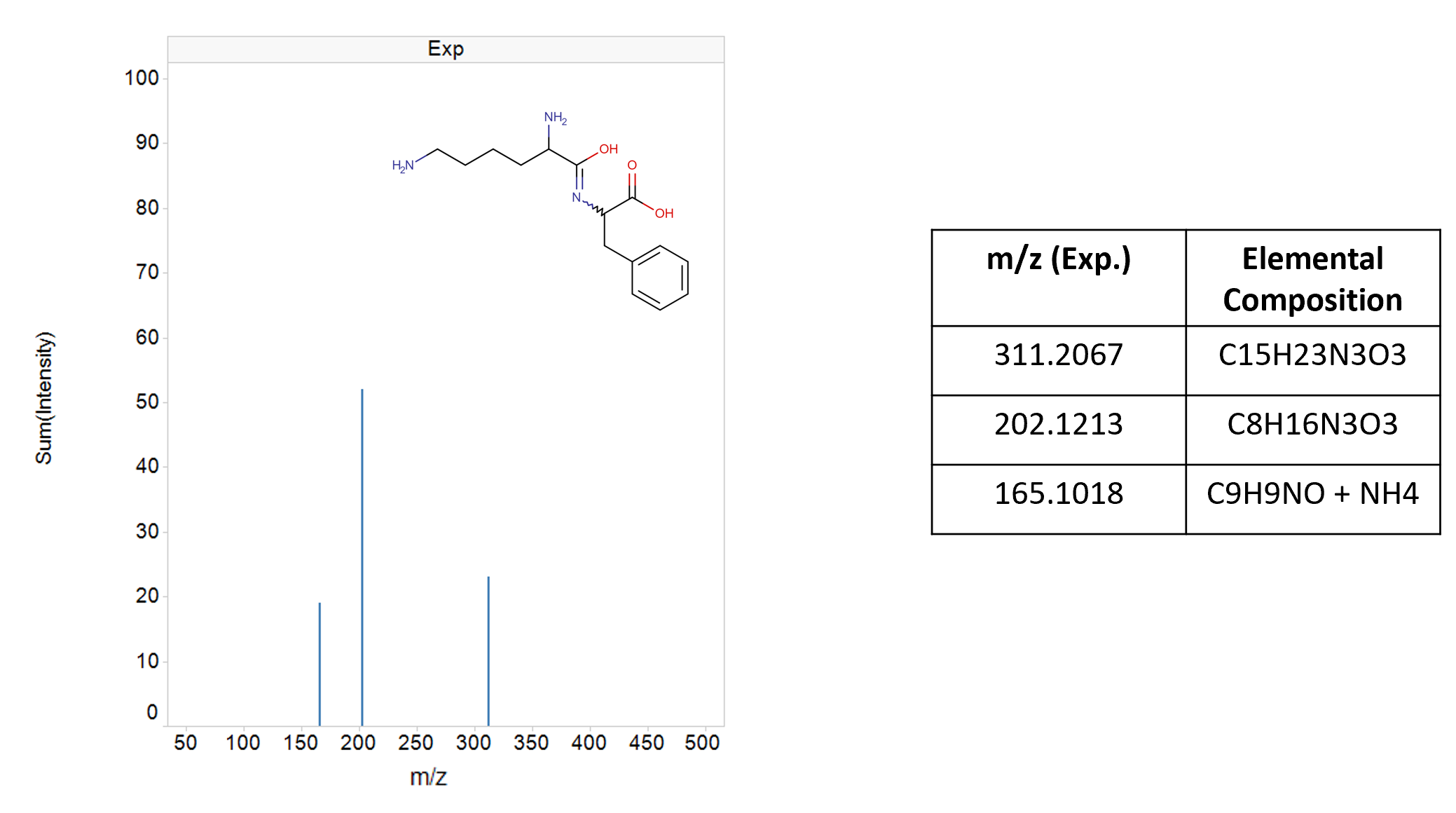


**Figure S9:** MS/MS spectra for lysylphenylalanine based on experimental data only (i.e., authentic standard commercially unavailable).


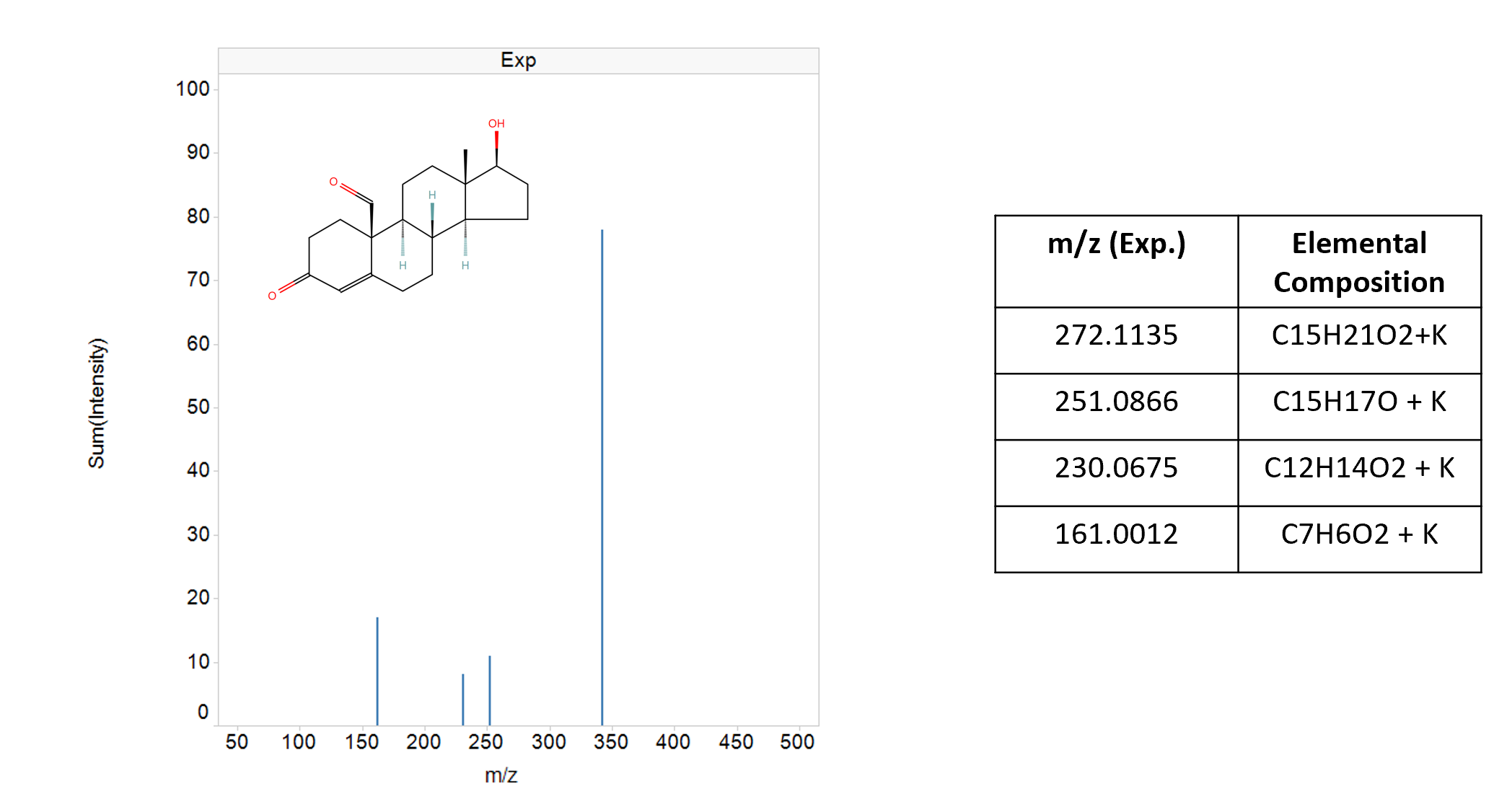


**Figure S10:** MS/MS spectra for 19-oxotestosterone based on experimental data only (i.e., authentic standard unavailable).


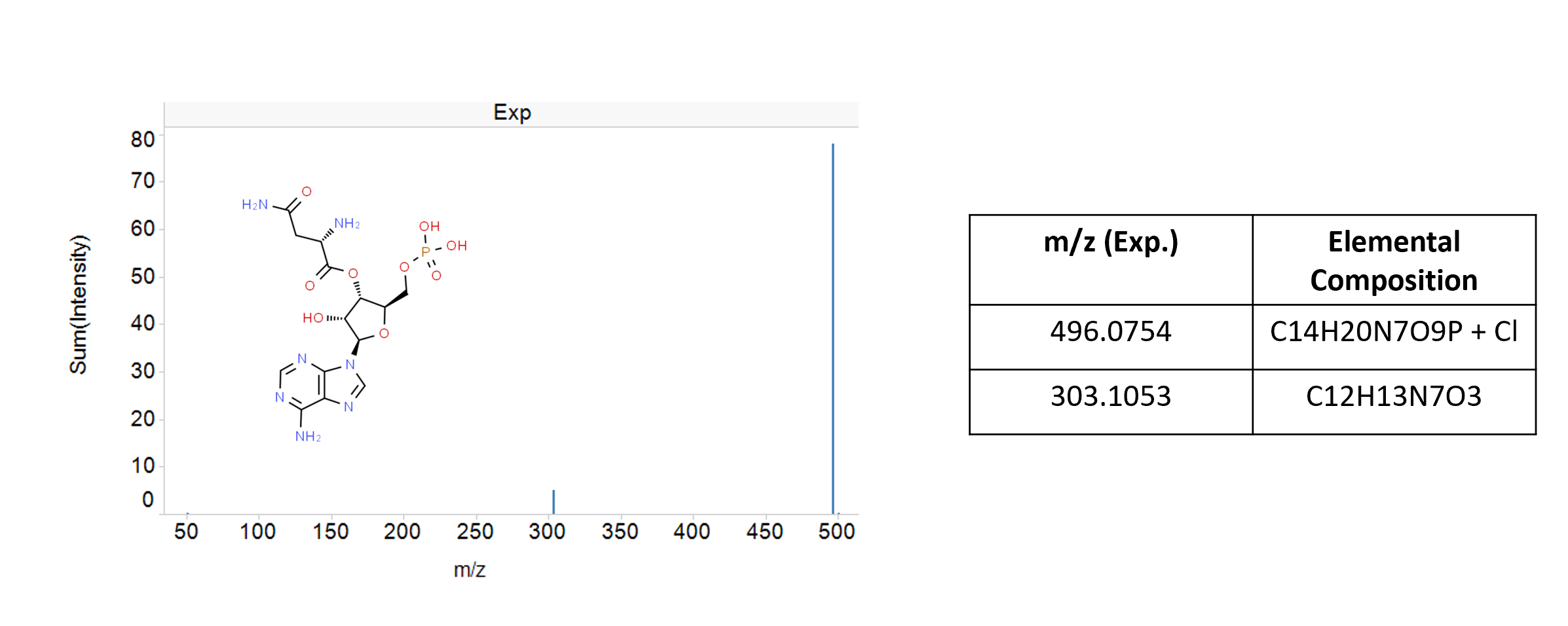


**Figure S11:** MS/MS spectra for 3’-L-asparaginyl-AMP based on experimental data only (i.e., authentic standard commercially unavailable).
